# Validation of an Adaptive Assessment of Executive Functions (Adaptive Cognitive Evaluation-Explorer): Longitudinal and Cross-Sectional Analyses of Cognitive Task Performance

**DOI:** 10.2196/60041

**Published:** 2025-04-21

**Authors:** Kristine D O'Laughlin, Britte Haugan Cheng, Joshua J Volponi, John David A Lorentz, Sophia A Obregon, Jessica Wise Younger, Adam Gazzaley, Melina R Uncapher, Joaquin A Anguera

**Affiliations:** 1 Neuroscape Department of Neurology University of California San Francisco San Francisco, CA United States

**Keywords:** executive functions, serious games, validation, computerized assessment, cognitive assessment

## Abstract

**Background:**

Executive functions (EFs) predict positive life outcomes and educational attainment. Consequently, it is imperative that our measures of EF constructs are both reliable and valid, with advantages for research tools that offer efficiency and remote capabilities.

**Objective:**

The objective of this study was to evaluate reliability and validity evidence for a mobile, adaptive measure of EFs called Adaptive Cognitive Evaluation-Explorer (ACE-X).

**Methods:**

We collected data from 2 cohorts of participants: a test-retest sample (N=246, age: mean 35.75, SD 11.74 y) to assess consistency of ACE-X task performance over repeated administrations and a validation sample involving child or adolescent (5436/6052, 89.82%; age: mean 12.78, SD 1.60 years) and adult participants (484/6052, 8%; age: mean 38.11, SD 14.96 years) to examine consistency of metrics, internal structures, and invariance of ACE-X task performance. A subset of participants (132/6052, 2.18%; age: mean 37.04, SD 13.23 years) also completed a similar set of cognitive tasks using the Inquisit platform to assess the concurrent validity of ACE-X.

**Results:**

Intraclass correlation coefficients revealed most ACE-X tasks were moderately to very reliable across repeated assessments (intraclass correlation coefficient=0.45-0.79; *P*<.001). Moreover, in comparisons of internal structures of ACE-X task performance, model fit indices suggested that a network model based on partial correlations was the best fit to the data (*χ*^2^_28_=40.13; *P*=.06; comparative fit index=0.99; root mean square error of approximation=0.03, 90% CI 0.00-0.05; Bayesian information criterion=5075.87; Akaike information criterion=4917.71) and that network edge weights are invariant across both younger and older adult participants. A Spinglass community detection algorithm suggested ACE-X task performance can be described by 3 communities (selected in 85% of replications): set reconfiguration, attentional control, and interference resolution. On the other hand, Pearson correlation coefficients indicated mixed results for the concurrent validity comparisons between ACE-X and Inquisit (*r*=–.05-.62, *P*<.001-.76).

**Conclusions:**

These findings suggest that ACE-X is a reliable and valid research tool for understanding EFs and their relations to outcome measures.

## Introduction

### Background

Executive functions (EFs) have been a fascination of researchers and educators alike due to their association with positive life outcomes and educational attainment. Understanding this complex set of cognitive processes that enables control over concentration and attention (the study by Diamond [1] presents a review on EFs) and their measurement by proxy is essential to furthering our collective knowledge of how they impact and interact with other important health and cognitive outcomes across the lifespan. To date, the field of EF research has largely relied on traditional laboratory-based tests designed to measure various aspects of EFs. However, these classic measures of EFs have been burdened by measurement challenges, such as in-person data collections, floor and ceiling effects, and measurement impurity related to using only one task to index an EF construct [2,3]. Moreover, in 2020, a global pandemic drastically altered our commonplace practices of conducting research and collecting data, forcing research endeavors out of the controlled laboratory setting and into more familiar and relaxed environments. This shift has pushed researchers to adopt remote designs conducive to conducting research anywhere, at any time. Furthermore, with remote data collection becoming the norm rather than the exception, researchers are tasked with developing innovative solutions to resolve discrepancies in outcomes observed in the laboratory versus in the real world.

Attempts to measure EFs have presented considerable challenges, with comparable difficulties experienced by those attempting to model them. While there is general agreement that the construct of EF is multidimensional [4-6], there has been much debate regarding the number and organization of these dimensions. For example, 1 study [7] indicated that as many as 18 components of EF have been suggested. Moreover, this organization is believed to shift over the course of development, as the differentiation hypothesis posits that EF represents a unitary construct during early childhood, eventually differentiating to 2 or 3 components in later childhood and adolescence [8,9]. Along with this, some have argued for the presence of a common EF component, which reflects shared commonality across all EF tasks [10,11]. In these models, EF task performances are associated with not only their hypothesized specific dimension of EF but also with common EF. Once common EF is accounted for, what should be left in theory are the dimension-specific associations within task performances.

While the confirmatory factor analysis approach has been the predominant method of understanding how latent EFs give rise to EF task performance, recent explorations have questioned whether these models are the best representation of EFs [12,13]. EF task performances tend to share a great deal of overlapping variance, and this shared variance is seldom adequately explained by correlating EF factors alone, as often significant correlations between EF tasks can be found even after correlating these factors. This would suggest reliable associations at the task level that do not correspond with a singular EF construct. Often, this shared task variance is reduced to the shared variance related to the method of testing rather than to the specific latent component of EF [5,14]. For example, a set of tasks in which both require participants to perform the same set of actions in reverse order are likely related due to the similarities in how the measurement occurred rather than to an underlying EF construct. In a factor analytic framework, this could be problematic, as leaving such correlations unmodeled could result in poor model fit due to misspecification, while modeling the correlation could create problems with identification and interpretation. Moreover, because of the reliance of more than one 1 EF in successfully executing tasks (eg, maintaining attention as a first requirement of executing a working memory task), task performances tend to be associated with more than one construct. While researchers have attempted to negate this shortcoming of factor analysis through modeling of a “common” or “general” EF construct [10], thereby separating construct-specific variance from general EF variance, in practice these models are often quite complex, and convergence issues often emerge [14].

We believe that a perhaps better conceptualization of EFs involves a network of task performances that directly relate to one another and can be decomposed into subsets, or “communities” of tasks. These task groupings can then provide evidence for or against the idea of internal validity of task organizations. When modeling EF task performance as a network using a partial correlation matrix, the correlation between each pair of task performances is represented as the association after all other task performances have been accounted for. In this way, each association of task performances represents shared variance beyond what is shared with the full set of tasks. Because the shared variance related across the entire set of tasks is accounted for, not only is the variance related to constructs accounted for (ie, common or general EF), but also the shared method variance that can occur across constructs. We also advocate for network analysis as it closely aligns with the neural networks that give rise to these processes, as meta-analytic results of functional magnetic resonance imaging data suggest that superordinate cognitive control systems as well as specific brain regions are engaged during execution of EF tasks (a study by Niendam et al [15] presents a review on the same). In theory, the network model would account for these superordinate processes by allowing the unique relations between task performances to be visualized. Moreover, using network analysis, EF task performances can be mapped in such a way so that the relative proximity to neighboring task performances reflect the degree of similarity between sets of tasks. This information can be helpful in establishing which EF task performances are most central within a set of tasks. Having this knowledge is potentially important in the context of intervention, as isolating tasks with the most potential for transfer to other EF performance could be particularly impactful for treatments aimed at improving EF skills. Therefore, we argue that this method, in combination with a community detection algorithm, allows for a more novel approach to answering the question of how EF tasks group together to form constructs.

### Adaptive Cognitive Evaluation-Explorer

Adaptive Cognitive Evaluation-Explorer (ACE-X) is a research tool developed at Neuroscape, a center in the Department of Neurology at the University of California, San Francisco, to assess the 3 facets of cognitive control (attention, working memory, and cognitive flexibility) [16,17]. However, this platform can also assess a breadth of EF dimensions, specifically the 3 most identified components of EFs (working memory, shifting, and inhibition), which aligns with the goal of being able to test the aforementioned theories of EF. Each ACE-X task is based on traditional EF measures but with key modifications that include engaging dialogue to better explain task instructions and features, high-level art, and music to create a more immersive participant experience, and most importantly, unique adaptive mechanisms for each module. These integrated adaptive algorithms mitigate persistent measurement issues, such as floor and ceiling effects, by adjusting the difficulty of each module in real time to an appropriate level of difficulty for the ability of the task taker. Moreover, these adaptive mechanisms not only benefit performance comparisons across groups but also facilitate comparisons of performance over time. Note that ACE-X has been designed to be an open-access technology that researchers, clinicians, teachers, or other interested parties can use to answer research-based questions.

ACE-X was adapted from its predecessor, Adaptive Cognitive Evaluation-Classroom (ACE-C) [13,18], which transformed standard EF measurement tools into an interactive and engaging user experience but lacked specific features that would facilitate self-administration in a remote setting. The primary enhancement associated with ACE-X from its ACE-C predecessor involved the incorporation of targeted gamification. Our development team focused their efforts toward augmenting trial-by-trial feedback, improving the in-game point system, and enhancing the art and music components. These facets were targeted to improve user motivation and engagement (the studies by Lumsden et al [19] and Vermeir et al [20] present reviews on the same). In a meta-analysis conducted by Lumsden et al [19], tasks that included gamification were rated more intrinsically motivating than similar tasks that did not. Moreover, when applied in a training context, gamified tasks have been shown to result in positive outcomes, such as improved cognitive control [21], mitigation of working memory declines associated with attention-deficit/hyperactivity disorder (ADHD) [22], and greater trial engagement [23] for some populations. Furthermore, these gamification elements may be more beneficial to certain populations than others: for example, Gallen et al [24] found that in their sample of adult participants, ADHD symptoms were negatively related to reward responsiveness, as those who reported having more ADHD symptoms were less responsive to reward. Moreover, age was negatively related to attention, such that younger participants tended to show greater improvements to attention when gamified features were present. Gamified tasks have also been shown to be valid representations of more traditional cognitive tasks [25-28]. For example, Aalbers et al [25] established good to very good convergent validity evidence in the domains of working memory, visuospatial short-term memory, and EF planning as demonstrated by relations between their gamified set of tasks (Brain Aging Monitor-Cognitive Assessment Battery) and other sets of tasks meant to tap the same domains. Furthermore, using exploratory factor analyses, McPherson and Burns [27] demonstrated that their gamified cognitive task set, Space Code, loaded onto task domains of working memory or fluid intelligence and processing speed. Thus, there is evidence that gamification of traditional tasks may improve participant engagement without negatively impacting validity, resulting in better participant outcomes for some populations.

It should be noted that other digital tools designed to assess EFs have their own strengths and limitations, just like the ACE-X battery. ACE-X is unique in that it specifically focuses on assessing cognitive control abilities in depth, unlike other commonly used batteries that aim to assess several different abilities, including language, long-term memory, eye movements, and logic, among others. Several aspects about ACE-X make it distinct from other technologies, including the aforementioned incorporation of adaptive mechanics and gamification of the testing experience. One of the innovative aspects of ACE-X is that it has been designed to be self-administered for individuals across the lifespan, whereas many of these other batteries need to be administered by a trained researcher and are only appropriate for specific, narrow age ranges. Another innovative aspect that makes ACE-X distinct from other tools is the ability for it to be used on cellular devices (iOS and Android), tablets (eg, iPad or Android devices), or web browsers. To further extend the scalability of ACE-X, data collection is possible when there is limited or no Wi-Fi connection (after having loaded the ACE-X app before testing), with any data collected uploaded to a secure cloud-based server the moment said device reconnects to Wi-Fi. Finally, one of the overarching goals for ACE-X was to have a tool with known test-retest values (which are not reported for many cognitive platforms) that could be used over multiple sessions, as its value is not only for single characterization efforts but also to act as an outcome measure for intervention studies.

### Overview of This Study

Here, we present reliability and validity evidence in support of ACE-X as a research tool for understanding EFs and their relations to external variables (eg, prediction of academic prowess and workplace achievement). Across 2 separate cohorts with >6000 participants, we first provide evidence of the reliability of task performance across time using a longitudinal sample of test takers. Second, using a cross-sectional sample of age-diverse test takers, we contrast ACE-X performance metrics against ranges observed in relevant literature. Next, we evaluated the task reliability within a single measurement session as well as the correspondence between ACE-X tasks and theoretical EF constructs using a combination of factor and network analyses (ie, validity of internal structures). Finally, we present associations between ACE-X tasks and a similar set of measures (ie, concurrent validity). We conclude with future research opportunities for ACE-X, as well as implications and recommendations regarding the use of ACE-X and its place in the current landscape of EF research.

## Methods

### Test-Retest (Longitudinal Study)

#### Study Design

Participants were recruited via Amazon Mechanical Turk (MTurk) for the test-retest study. The following selection criteria for MTurk participants were included: (1) must be located in the United States, (2) must have completed ≥50 approved human intelligence tasks, and (3) must have ≥95% of assignments approved. Sample size for the test-retest study was determined via power analysis, which suggested approximately 84 participants for each of 2 possible combinations of ACE-X counterbalanced subsets were needed to achieve sufficient power to detect a moderate effect size; therefore, we looked to enroll 200 participants (100 per task set) with at least 2 playthroughs of the task set combinations. Recruitment and data collection for the test-retest sample took place simultaneously between January 2020 and July 2020. In total, 533 participants completed the first playthrough of ACE-X. Of these, 47.6% (254/533) went on to complete at least one additional playthrough of ACE-X counterbalanced subsets, with the average duration between assessments being 7.29 (SD 15.82) days. Participants who completed the second assessment session later than 31 days after the first session were excluded from analysis (8/533, 1.5% participants excluded). After removing these participants, the average duration between assessments was 4.90 (SD 6.31) days. These data were also cleaned for anticipatory responses (reaction time<200 ms; 39% of trials removed) and trials that were beyond 3 individual SDs of the individual mean response time (1.1% of trials removed). Next, data were removed for having <5 trials per task condition (average of 1.55% of scores removed per task) and for performance less than chance accuracy (average of 0.77% of scores removed per task). Finally, outliers beyond ±3 median absolute deviations of the median of scores for that assessment session were removed on a task-by-task basis (average of 4.06% of scores removed per task). The resulting dataset included 46.2% (246/533) participants with at least 2 playthroughs of ACE-X subsets.

#### Ethical Considerations

All study procedures were conducted in accordance with protocols approved by the institutional review board at the University of California, San Francisco (IRB 19-28330). Written informed consent was obtained before study participation. This written documentation clearly stated that participants were allowed to opt out of the study at any time and with no penalty other than loss of study benefits. Participants were compensated US $10 for their participation. No potentially identifying information other than the MTurk user’s unique identification number and participant age was collected; therefore, no other deidentification procedures were required.

#### Measures

##### Adaptive Cognitive Evaluation-Explorer

**Figure 1:**
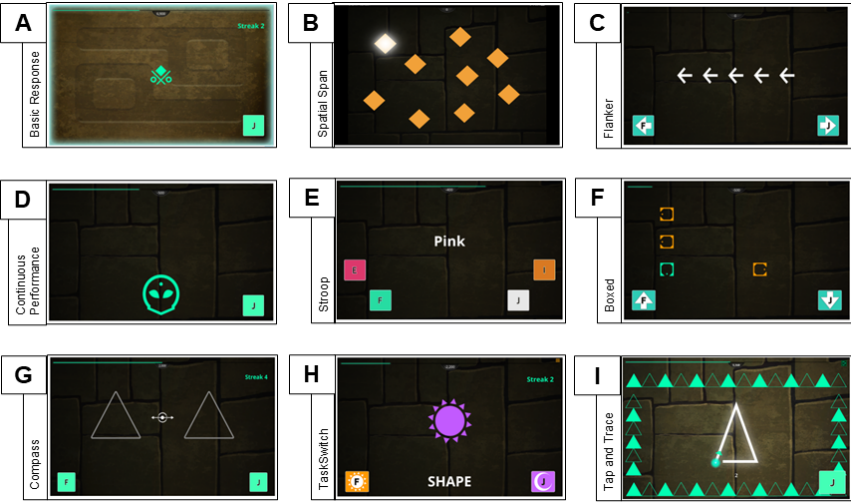
Example task schematics for Adaptive Cognitive Evaluation-Explorer (ACE-X). Panel (A) shows the Basic Response Time task, (B) shows the spatial span task (forward span trial), (C) shows the Flanker task (congruent condition), (D) shows the continuous performance task (no-go trial), (E) shows the Stroop task (incongruent condition), (F) shows the Boxed task (feature 4 condition), (G) shows the Compass task (neutral condition), (H) shows the TaskSwitch task (incongruent condition), and (I) shows the Tap and Trace task (dual task condition).

ACE-X includes 1 task measuring general processing speed, 2 tasks meant to measure working memory or short-term memory, 6 tasks measuring inhibitory control, and 2 tasks measuring cognitive flexibility. Example task schematics for ACE-X are shown in Figure 1.

##### Adaptivity

Most ACE-X tasks adapt through a closed-loop mechanism on a trial-by-trial basis, which modifies a participant’s adaptive response window (subsequent sections present exceptions) by either increasing or decreasing the length of time that a participant has to make a response that is considered “correct and on time.” The algorithm uses a “one-up-four-down” approach [29], where the length of the response window decreases by a step factor of 10ms when the response is correct and increases by a step factor of 40 ms when the response is incorrect or late. After each response, the response window is adjusted by –(10 ms×2^ [previous consecutive correct trials]) for each successive correct and on-time response, or by +(40 ms×2^ [previous consecutive incorrect trials]) for each successive incorrect or late response. This allows the response window to rapidly adapt in real time to the individual test-taker’s ability. This adaptive response window is paired with feedback after each trial indicating whether the response was incorrect (red), correct but late (yellow), or correct and on time (green) to help participants monitor and adjust their rate of responding. This design encourages participants to balance both speed and accuracy in responding to EF task demands.

The adaptive features of ACE-X provide a mechanism for adjusting task difficulty based on the cognitive control abilities of the test taker; thus, ACE-X scales on a trial-by-trial basis based upon the performance of the test taker, regardless of demographics. Tasks with similar adaptive mechanisms were shown to be efficient and reliable embodiments of classic cognitive tasks [21,30,31]. For example, Draheim et al [31] compared adaptive versions of Flanker [32] and Stroop [33] tasks to nonadaptive versions of these classic cognitive assessments. In the adaptive versions of these tasks, participants were given a “response deadline,” which allotted a maximum time limit in which they could register a response to the trial. This response deadline became longer when participants responded incorrectly or too slowly with respect to the response deadline and shorter when they responded correctly. Their findings suggested that the adaptive versions performed better than the classic assessments in terms of test-retest reliability, average attention factor loading, and correlations with working memory capacity and fluid intelligence. Therefore, at least in some circumstances, adding adaptive algorithms to traditional task designs appears to improve task psychometrics.

##### Processing Speed

General processing speed was measured via the Basic Response Time task. In this task, participants were instructed to respond by selecting a button as quickly as possible every time the target symbol appeared in the center of the screen (Figure 1A). Participants first completed the task using their right hand, followed by their left hand. The overall mean response time across performance for both hands was used as a control for tasks requiring 2-hand responding, while mean response time for the dominant hand was used as a control for tasks requiring 1-hand responses.

##### Working Memory

Forward and backwards spatial span (Gem Chaser and Gem Chaser Backwards) were designed to measure visuospatial short-term and working memory capacity, respectively. On the basis of the Corsi block task [34], participants were shown an array of diamonds, with each lighting up one at a time to show the target sequence. Participants were asked to recall the sequence in the same (forward spatial span) or reverse order (backwards spatial span; Figure 1B). Sequence length started at 3 objects, and length increased by 1 each time the participant successfully recalled 2 sequences in a row, with possible sequence length ranging from 3 to 9. When a participant was unable to recall 3 sequences in a row, the task was terminated. For these tasks, we used object span, or the length of the longest sequence attempted in 2 consecutive trials by the participant, as our measure of visuospatial short-term and working memory capacity.

##### Inhibitory Control

The Flanker task (Flanker Arrow) is a measure of selective attention and interference resolution. On the basis of the original Flanker task [32], participants were shown a string of 5 arrows and asked to identify the direction (left or right) of the center (target) arrow (Figure 1C). In the congruent condition, the target arrow appeared in the same direction as the flanking arrows, whereas in the incongruent condition, the target arrow appeared in the opposite direction. For this task, the overall rate correct score (RCS) collapsed across conditions was used to index task performance [35,36]. RCS is computed as the number of correct responses divided by the product of the number of trials and total response time. RCS represents the average number of correct responses over 1 second.

Impulsive (Mars UFO) and sustained attention (Venus UFO) were measured using the continuous performance task (CPT), based on the test of variables of attention (TOVA) [37]. Participants were instructed to hit a button every time a symbol was displayed at the top of the screen (target) and do nothing when the symbol appeared at the bottom of the screen (distractor; Figure 1D). In the impulsive condition, the target appeared on the top portion of the screen in 80% of trials, while in the sustained condition, the target only appeared in 20% of trials. Because the CPT was meant to assess the ability to maintain attention, this task did not include an adaptive response window to avoid including elements that might inadvertently impact attention-directing abilities. Because a response was not required for every trial, performance was measured as the average response time to correctly answered trials separately for impulsive and sustained attention, rather than RCS as in other tasks.

The Stroop task (Color Tricker) was designed to measure response inhibition. In the Stroop task, as described by Stroop [33] and adapted by Mead et al [38], participants were shown a color spelled out in words and written in a particular ink color. Participants were asked to indicate the color of the text while ignoring the color the word spells (Figure 1E). For example, if the participant was shown the word WHITE written in green ink, the correct response would be *green*. There were a total of 4 response options for the Stroop task: pink, green, white, and orange. In congruent trials, the color of the word and the written word were the same, while in incongruent trials the color of the word and the word spelled by the text were different. Overall, RCS was used to measure performance on the Stroop task.

Boxed is a visual search task based on the paradigm described by Treisman and Gelade [39]. Participants were shown an array of Landolt squares with openings on 1 of the 4 sides (Figure 1F). Participants were instructed to attend to the single green square with a top or bottom opening (target) and ignore orange squares and squares in which the opening was not on the top or bottom (distractors). On each trial, participants were instructed to select the location (top or bottom) of the opening of the target square. In the feature conditions, the target and distractor boxes differed by a singular feature (color), while in the conjunction conditions they differed by a conjunction of features (color and location of opening). There were 4 blocked conditions for this task, starting with an array of 4 squares differing only in feature up to 12 boxes differing in both color and location of opening. Overall RCS was again used as the metric of interest for this task.

The Compass task is a measure of selective attention based on the Posner cueing task [40]. In the Compass task, participants were shown 2 blank triangles on the right and left sides of the display (Figure 1G). An arrow appeared in the center of the display pointing toward either the right triangle, the left triangle, or both triangles. After 500 ms, a symbol appeared in one of the triangles, and participants were instructed to select the side in which the symbol appeared, regardless of the direction the arrow pointed. In neutral trials the arrows pointed at both triangles, in congruent trials the arrow pointed at the triangle where the symbol appears, and in incongruent trials the arrow pointed at the opposite triangle of the one displaying the symbol. Overall RCS was also used to measure performance on the Compass task.

##### Cognitive Flexibility

TaskSwitch (Sun & Moon) is based on standard task-switching paradigms (the study by Monsell [41] presents a review on the same). In this task, participants were shown a cue indicating a feature that the participant should attend to (either color or shape), followed by a stimulus (eg, purple moon; Figure 1H). Participants were instructed to select the cued feature of the stimulus. For example, if the cue was “shape,” followed by an orange sun, the participant should have selected “sun“ to make a correct response. On stay trials, the cued aspect was the same as the previous trial, whereas on “switch” trials the cued aspect was different from the previous trial. For this task, overall RCS was again used as the main performance measure.

Finally, the Tap and Trace (Triangle Trace) task was adapted from the dual-task paradigm of Eversheim and Bock [42] and Anguera et al [21]. In the first block of the task, participants were instructed to press a button when the screen was bordered by green triangles (target) while doing nothing in response to all other colors or shapes (distractors; eg, brown rectangles; Figure 1I). In the second block, participants were asked to multitask; in addition to performing the shape detection task, participants were instructed to simultaneously trace a figure with the nondominant hand. Finally, in the third block, participants were asked only to trace figures with their nondominant hand. Multitasking ability was measured using the mean response time to correct trials during the multitasking block.

#### Data Analysis

We assessed test-retest reliability by examining intraclass correlation coefficients (ICCs) between first and second ACE-X assessment sessions. In assessing test-retest reliability, we judged ICCs >0.75 as excellent, between 0.60 and 0.74 as good, and between 0.40 and 0.59 as fair [43]. This analysis included all 246 participants completing at least 2 sessions of ACE-X as part of the test-retest cohort.

### ACE-X Validation (Cross-Sectional Study)

#### Study Design

The sample size for the ACE-X validation study was determined using a combination of 2 approaches to ensure findings were both representative of the population at large and properly powered for statistical purposes. The first approach focused on ensuring sufficient representation across age groups, ethnicities, and socioeconomic status. The second approach focused on having sufficient power in computing correlations to assess concurrent validity. For the first approach, our goal was to have 100 participants per target age bracket (each year from 7-18 years, 19-27 years, 28-36 years, 37-45 years, 46-54 years, 55-63 years, and ≥64 years). Within each age bracket, participants were recruited according to nationally representative proportions of poverty and ethnicity categorizations. For the second approach, power analyses revealed that a minimum of 84 participants would be needed to establish 80% power for a moderate correlation. Therefore, a goal of 100 participants was established for assessing concurrent validity. However, recruitment streams remained open during the duration of the grant cycle, and ultimately, 9275 participants were recruited for the ACE-X validation study. Of these, 89.88% (8336/9275) completed only ACE-X as contributors to a normative database of task performance, while a smaller number (939/9275, 10.12%) completed both ACE-X and a second set of tasks meant to establish relationships with other variables (concurrent validity). Study recruitment and data collection for the ACE-X validation study took place simultaneously between May 2021 and January 2023. Adult participants were recruited via advertising on various social media platforms (eg, Craigslist [Craig Newmark] and Facebook [Meta Platforms]), MTurk (Amazon, Inc), and a Qualtrics (Qualtrics International Inc) recruitment panel. Child participants were primarily recruited through partnerships with local schools and the Character Lab Research Network, though some came through other social media advertisements. For those who found the study via social media, upon engaging with an advertisement by clicking on a link, participants were directed to a web-based platform to complete registration, consent, and eligibility screening. Eligible participants then completed a questionnaire and ACE-X. A subset of these participants also completed a second suite of tasks measuring various aspects of EFs (Inquisit; refer to the subsequent sections). Forced breaks were imposed during the testing session to mitigate participant fatigue.

All data were cleaned for anticipatory responses (0.77% of trials removed) and trials that were beyond 3 individual SDs of the individual mean response time (1.16% of trials removed). Next, data were removed for having fewer than 5 trials per task condition (average of 13.1% of scores removed per task) and for performance less than chance accuracy (average of 3.28% of scores removed per task).

Due to the fully remote data collection design of this study, data originating from social media, MTurk, and Qualtrics recruitment streams yielded a high proportion of suspicious or abnormal responses and were, therefore, carefully screened for potential bad actors. After extensive discussion among our research group and observing that a large proportion of these abnormal responses were completed from locations outside of the United States, originated from the same IP addresses, or made use of disposable email addresses, we labeled these as *high risk* indicators of false responding and opted to automatically exclude the participant if any of these were present (2887/9275, 31.13% excluded). Other responses to demographic questions were checked for consistency, and inconsistencies (eg, discrepancies between reported age and birthdate or between location and zip code information) were flagged as *moderate risk* but did not necessarily result in the participant’s immediate exclusion. However, after comparing the number and characteristics of the participants with ≥4 *moderate risk* indicators, we also decided to exclude these participants even in the absence of any *high risk* indicators (66/9275, 0.71% excluded). All remaining records were carefully screened by a team of researchers to determine the authenticity of all data included. Data for participants suspected of providing inauthentic responses were removed before analysis (270/9275, 2.91% excluded; a study by O’Laughlin [44] and Multimedia Appendix 1 presents more information about participant removal criteria). After removing these participants, outliers beyond –3 or +3 median absolute deviations of the median of scores were removed on a task-by-task basis (average of 3.79% of scores removed per task). This cleaning process resulted in a final sample size of 6052 (616/6052, 10.18% adult participants and 5436/6052, 89.82% child participants), of which 132 (2.18%) adult participants also completed Inquisit for concurrent validity analyses.

#### Ethical Considerations

Participants were compensated via electronic gift cards for their contribution. Initially, respondents were compensated US $5 upon completion of all study requirements; this amount was later increased to US $10 to encourage a higher participation rate. Data were deidentified before analysis.

#### Measures

##### Adaptive Cognitive Evaluation-Explorer

All 616 adult participants received the full set of 11 ACE-X tasks (described in detail in the Test-Retest [Longitudinal Study] section earlier), while all 5436 child participants received limited subsets of between 3 to 4 ACE-X tasks. Due to time constraints within classrooms, child or adolescent participants did not receive the full set of ACE-X tasks but instead completed a subset of tasks, including Basic Response Time and tasks corresponding to 1 of 3 hypothesized EF constructs. To avoid speeded responses on ACE-X tasks, we tested how long each subset of tasks took to complete, as well as reduced the number of surveys and moved remaining surveys to the end of the testing session to allow ample time to complete ACE-X tasks. We also ensured ≥2 orders for these task subsets of only 3 to 4 tasks to avoid order effects as much as possible. Adult participants who completed the study as part of the norming or concurrent validity arms received the full set of ACE-X tasks in a counterbalanced order.

##### Inquisit

One of the overarching goals of this study was to test for concurrent validity against *gold standard* validated measures of EF; however, the practical logistics of testing ACE-X in person against validated instruments was not tenable, especially during the COVID-19 pandemic. Thus, we opted to make comparisons to a similar set of measures that could be remotely administered, a suite of cognitive tasks known as Inquisit. Inquisit and ACE-X are similar in that both are mobile versions of traditional EF assessments. However, unlike ACE-X, Inquisit tasks do not adapt via response window, with Inquisit tasks more closely mirroring traditional “gold standard” measures of EFs. For each corresponding task, the same metric was used for both ACE-X and Inquisit tasks. ACE-X and Inquisit task comparisons are shown in Table S1 in Multimedia Appendix 1.

In Inquisit, general processing speed was measured via the Simple Visual Reaction Time task. Short-term memory and working memory were measured via the forward and backwards Corsi Block Tapping task [34,45]. Inhibitory control was measured via the Letter Flanker task [32]; the TOVA [37,46]; the classic Stroop task [33]; the Visual search task [47]; and the Cueing task [40]. Finally, cognitive flexibility was measured by the Category Switch task [48,49] and the Trail Making task [50,51].

For the Inquisit set of tasks, we completed the same cleaning steps as described previously. Data were cleaned for anticipatory responses (4.04% of trials removed) and trials that were beyond 3 individual SDs of the individual mean response time (0.87% of trials removed). Next, data were removed for having fewer than 5 trials per task condition (average of 11.09% of scores removed per task) and for performance less than chance accuracy (average of 5.71% of scores removed per task). After removing suspicious participants, outliers beyond –3 or +3 median absolute deviations of the median of scores were removed on a task-by-task basis (average of 8.28% of scores removed per task).

#### Data Analysis

##### Data Preparation

General processing speed was controlled using residualized scores after accounting for Basic Response Time. For tasks where mean correct response time was the metric of interest (CPT-TOVA; Tap and Trace-Trail Making), scores were multiplied by –1 and divided by 100 so that higher scores indicated better performance and to minimize extreme variance estimates.

##### Consistency of ACE-X Performance Metrics

To assess consistency between ACE-X performance metrics and those reported in similar studies across the literature, for each ACE-X task we examined a sampling of comparable studies to determine ranges of reported metrics (Table S2 in Multimedia Appendix 1 provides more information on selected references). Here, we examined the metrics most reported for each task, and where available, we provide minimum and maximum response time, accuracy, and object span (forward and backwards spatial span). This literature review was not meant to be exhaustive but rather meant to encompass studies reporting tasks most similar to ACE-X tasks and across similar subsets of age ranges (discrepancies in age ranges reported in the Results section). All ACE-X validation cohort data, including both the children and adults (6052 participants), were included in these comparisons.

##### Internal Structures

To understand the internal structure of ACE-X for adult participants, we began by taking an exploratory approach by fitting a network model using the psychonetrics package in R (R Foundation for Statistical Computing). We fit the partial correlation network model using full information maximum likelihood estimation to handle missing values and then pruned edge weights that failed to reach statistical significance (*P*>.05). We then used the Spinglass community detection algorithm [52] with 1000 replications to determine whether ACE-X tasks tend to form communities of similar EF skills. The Spinglass algorithm was selected over other common community detection algorithms (such as Louvain or modularity) due to its ability to handle negative edge weights (if any emerged).

After establishing the data-driven factor structure using network analysis, we used the communities found in the network model to guide confirmatory testing of ACE-X internal structures. We compared the network model to both a correlated factor model of EF and a bifactor model of EF. We used *χ*^2^ model fit statistics, along with the comparative fit index (CFI), the root mean square error of approximation (RMSEA), the Bayesian information criterion (BIC), and the Akaike information criterion (AIC) to assess how well the theoretical factor structures corresponded with the observed data. Lower *χ*^2^ values suggest a better fit to the data, with a nonsignificant result suggesting equivalence between the predicted model and the data. Because *χ*^2^ is known to be sensitive to sample size, we also evaluated fit indices of RMSEA and CFI. RMSEA values ≤0.06 were considered adequate model fit [53], with lower values indicative of a better-fitting model. CFI values >0.90 were considered excellent model fit, with values closer to 1 indicating better model fit. For BIC and AIC, lower comparative values were taken as indicative of better model fit. In analyses of internal structure and invariance (described in the subsequent sections), all adult participants’ data from the ACE-X validation cohort (616/6052, 10.18% participants) were included.

##### Invariance

We selected the best-fitting model based on the criteria described earlier to then examine invariance between younger (aged between 18 and 39 years) and older (aged ≥40 years) adults. We examined whether applying equality constraints to loadings (weak invariance), intercepts (strong invariance), and residual variances (strict invariance) resulted in detriments to model fit such that models are found to vary between groups. We compared nested models using *χ*^2^ likelihood ratio tests with df equal to the difference in the number of free parameters between the comparison and nested models.

##### Relations to Other Variables

Finally, we assessed relationships with other variables by examining Pearson correlations between ACE-X and Inquisit for the subset of adult participants completing both sets of tasks (132/6052, 2.18%). We used correlation sizes of 0.10, 0.30, and 0.50 to indicate small, moderate, and large effects, respectively [54]. These values were then used as indicators of weak, moderate, and strong concurrent associations between pairs of EF tasks. For this analysis, all 132 participants completing both ACE-X and Inquisit as part of the ACE-X validation cohort were included.

## Results

### Test-Retest (Longitudinal Study)

The mean age of participants meeting the selection criteria for the test-retest study was 35.75 (SD 11.74) years. Test-retest reliability as ascertained by ICCs for the test-retest sample of participants suggests good to excellent reliability for most ACE-X tasks (Table 1). Boxed and Compass were considered exceptionally reliable, while good reliability was also achieved for Basic Response Time, Flanker, both CPTs, Stroop, TaskSwitch, and Tap and Trace. The only tasks to fall below this threshold were forward and backwards spatial span, perhaps due to the restricted range of the outcome metric for this set of tasks (this is described in detail in the Discussion section), though reliability would still be considered fair for these tasks. Overall, these findings suggested that ACE-X tasks can be considered consistent across testing sessions that take place about a week apart.

**Table 1:** Intraclass correlation coefficients for test-retest reliability of Adaptive Cognitive Evaluation-Explorer (ACE-X) tasks.

Task	ICC^a^	*P* value
Basic response time	0.70	<.001
Forward spatial span	0.45	<.001
Backwards spatial span	0.54	<.001
Flanker	0.62	<.001
Continuous performance (impulsive)	0.66	<.001
Continuous performance (sustained)	0.63	<.001
Stroop	0.71	<.001
Boxed	0.79	<.001
Compass	0.77	<.001
TaskSwitch	0.66	<.001
Tap and trace	0.70	<.001

^a^ICC: intraclass correlation coefficient.

### ACE-X Validation (Cross-Sectional Study)

#### Participant Characteristics

Participant demographics for each arm of the ACE-X validation study are shown in Table 2.

**Table 2:** Demographics for normative databases and concurrent validity samples.

Demographic		Normative data sample (ACE-X^a^ only)			Concurrent validity sample (ACE-X+Inquisit)
		Child	Adult	Adult	
**Gender, n (%)**					
	Woman	2415 (45.85)	236 (58.9)	68 (51.5)	
	Man	2573 (48.85)	155 (38.7)	62 (47)	
	Nonbinary or other	139 (2.64)	9 (2.2)	2 (1.5)	
	Prefer not to answer	140 (2.66)	1 (0.3)	0 (0)	
	Total reporting gender	5267 (100)	401 (100)	132 (100)	
**Origin, n (%)**					
	Hispanic	2392 (44.26)	68 (14.1)	45 (34.1)	
	Non-Hispanic	2919 (54.01)	412 (85.1)	87 (65.9)	
	Prefer not to answer	94 (1.74)	4 (0.8)	0 (0)	
	Total reporting origin	5405 (100)	484 (100)	132 (100)	
**Ethnicity, n (%)**					
	Asian or Pacific Islander	495 (9.11)	76 (15.7)	21 (15.9)	
	Black or African American	1036 (19.06)	59 (12.2)	31 (23.5)	
	Native American	29 (0.53)	6 (1.2)	2 (1.5)	
	White	3527 (64.88)	278 (57.4)	47 (35.6)	
	≥2 ethnicities	138 (2.54)	25 (5.2)	7 (5.3)	
	Not listed	2 (0.04)	0 (0)	0 (0)	
	Prefer not to answer	209 (3.84)	40 (8.3)	24 (18.2)	
	Total reporting ethnicity	5436 (100)	484 (100)	132 (100)	
**Free or reduced price lunch, n (%)**					
	Yes	2447 (46.63)	—^b^	—	
	No	2801 (53.37)	—	—	
	Total reporting free or reduced price lunch	5248 (100)	—	—	
Age (y), mean (SD)		12.78 (1.6)	38.11 (14.96)	37.04 (13.23)	
Income (US $), mean (SD)		148,799 (116,773)	78,220 (77,865)	58,429 (90,911)	
Total, n (%)		5436 (100)	484 (100)	132 (100)	

^a^ACE-X: Adaptive Cognitive Evaluation-Explorer.

^b^Not applicable.

#### Consistency of ACE-X Performance Metrics

Tables S3-S11 in Multimedia Appendix 1 present ACE-X mean performance metrics by age group, while Table 3 provides minimum and maximum mean values as reported in a selection of EF literature (Table S1 in Multimedia Appendix 1 provides more information on selected references). The majority of ACE-X tasks show mean values that fall within observed ranges, with a few exceptions. Notably, for the Flanker task, mean response times were slower than those observed in the selection of relevant literature for ages ≤17 years and ≥40 years. However, these age groups were absent from our literature review, making it unclear whether these response times are typical for the youngest and oldest participants in our sample. Moreover, these values were not extremely different from those observed in the literature (2%-11% slower than the literature reported maximum value); therefore, while outside of the observed ranges with other age groups, differences were mostly negligible when compared to ranges for those ages. Two tasks also showed consistent differences in accuracy. Accuracy for TaskSwitch was consistently lower for all reported age groups. However, the accuracy was higher on the Tap and Trace task for all but the ≤12 years age group. These differences may be due in part to ACE-X’s adaptive mechanisms, which are meant to balance speed and accuracy. Furthermore, the unique task design of Tap and Trace may have made this task less comparable to other tasks in the selected literature, thus yielding differences in accuracy.

**Table 3:** Minimum and maximum values of similar tasks performance metrics reported in the selected literature.

Tasks and metrics			Values, range
**Basic response time**			
	Response time	231-518	
**Forward spatial span**			
	Object span	4.80-9.61	
**Backwards spatial span**			
	Object span	4.20-9.05	
**Flanker**			
	Response time	350-569	
	Accuracy (%)	89-100	
**Continuous performance (impulsive)**			
	Response time	294-460	
**Continuous performance (sustained)**			
	Response time	346-626	
**Stroop**			
	Response time	594-818	
	Accuracy (%)	84-98	
**Boxed**			
	Response time	422-2250	
	Accuracy (%)	89-99	
**Compass**			
	Response time	267-1075	
**TaskSwitch**			
	Response time	500-1440	
	Accuracy (%)	92-100	
**Tap and trace**			
	Response time	360-1280	
	Accuracy (%)	92-95	

#### Internal Structures

**Figure 2:**
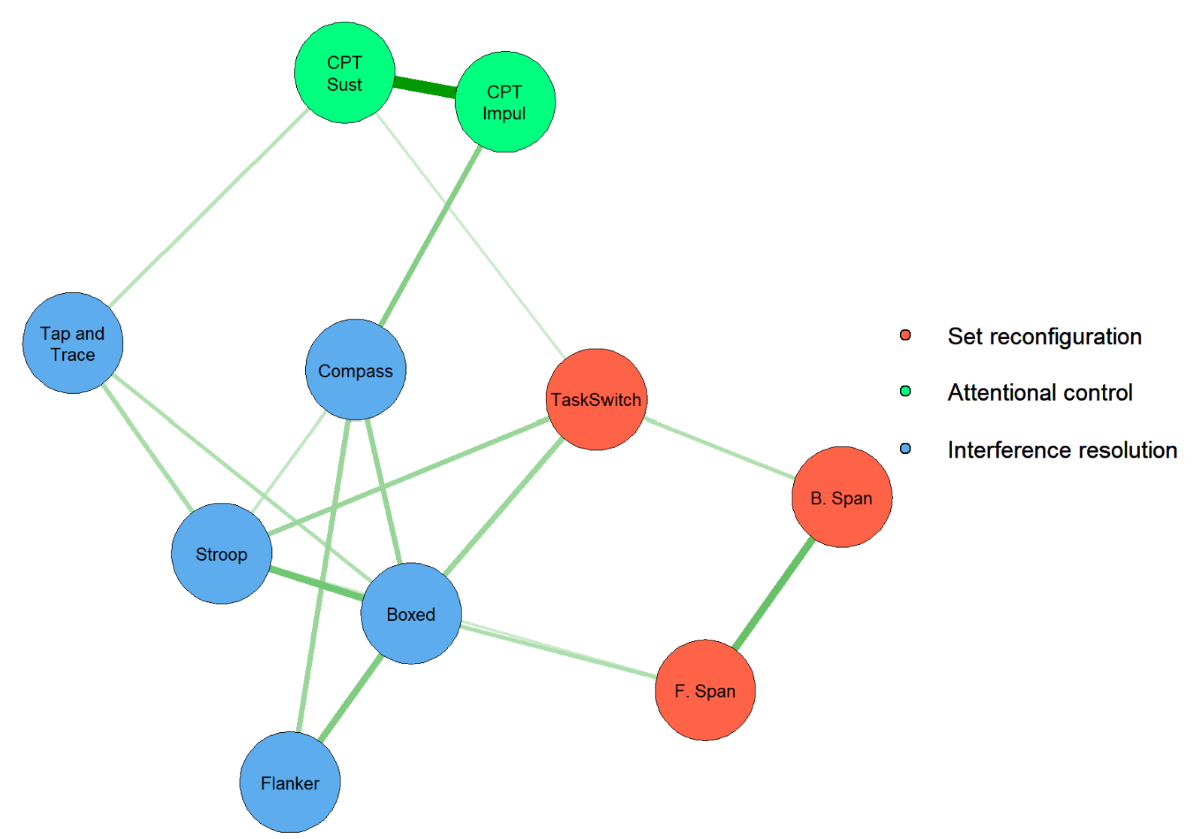
A network graph of Adaptive Cognitive Evaluation-Explorer (ACE-X) task performance. Network configuration was selected in 85% of Spinglass algorithm replications. B. Span: backwards spatial span; CPT: continuous performance task; F. Span: forward spatial span; Imp Attn: continuous performance task—impulsive; Sust Attn: continuous performance task—sustained.

After establishing the reliability and consistency of performance metrics with relevant literature, we found support for 3 communities of ACE-X task performances using network analysis. Results of this analysis and community detection performed using adult participants from the ACE-X validation cohort are shown in Figure 2 (Table S12 in Multimedia Appendix 1 gives estimates and SEs of edge weights). After pruning nonsignificant partial correlations, 37 parameters (17 edge weights, 10 means, and 10 scalings) were estimated in this model, leaving 28 df. From these results, we suggest that the 3 communities corresponded to “set reconfiguration” comprised forward and backwards spatial span and TaskSwitch; “attentional control” comprised the 2 CPTs; and “interference resolution” was associated with Tap and Trace*,* Compass, Stroop, Boxed, and Flanker. This community configuration was selected in 85% of replications of the Spinglass algorithm. The strongest associations, as indicated by line thickness in the network graph, were observed between the 2 CPTs, between forward and backwards spatial span, between Stroop and Boxed, and between Boxed and Flanker. While these tasks were more strongly connected, other tasks, such as TaskSwitch, Tap and Trace, Compass, Boxed, and Stroop, were more distally related but shared connections with multiple communities of tasks. Of these tasks, TaskSwitch was the only one to share connections with all 3 communities, suggesting that switching abilities may be important for set reconfiguration as well as for attentional control and interference resolution (Figure S1 in Multimedia Appendix 1 depicts network centrality measures). Results of bootstrapped CIs suggested stability of edge weights across 2500 resamples and are presented in Table S13 in Multimedia Appendix 1.

**Figure 3:**
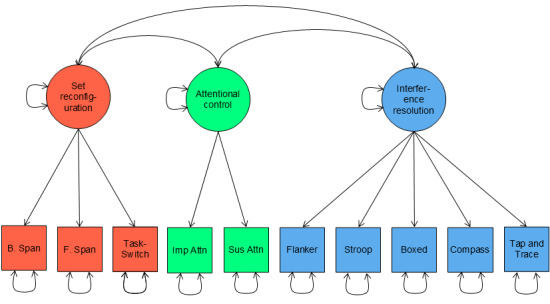
Path diagram of the correlated 3-factor model of Adaptive Cognitive Evaluation-Explorer (ACE-X) task performance. B Span: backwards spatial span; F Span: forward spatial span; Imp Attn: continuous performance task—impulsive; Sust Attn: continuous performance task—sustained.

**Figure 4:**
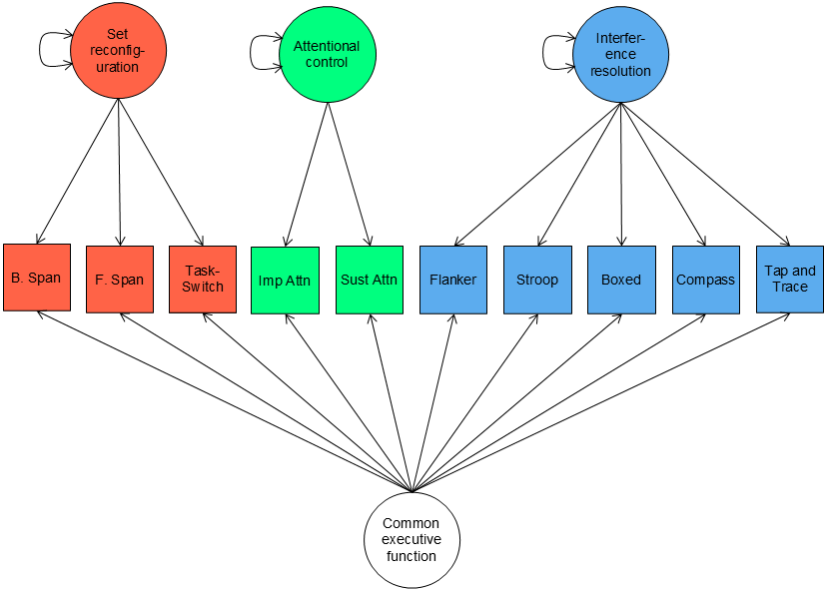
Path diagram of the bifactor model of Adaptive Cognitive Evaluation-Explorer (ACE-X) task performance. B Span: backwards spatial span; F Span: forward spatial span; Imp Attn: continuous performance task—impulsive; Sust Attn: continuous performance task—sustained.

On the basis of the results of the network analysis and community detection, we fit correlated 3-factor and bifactor models of EF to reflect factors of set reconfiguration, attentional control, and interference resolution. This approach revealed that while a correlated 3-factor model suggested ACE-X tasks are strong indicators of the underlying factors, the bifactor model may be overly complex in describing this set of data, as indicated by issues with convergence. Table 4 provides factor loadings for the correlated 3-factor and bifactor models of EF (factor models shown in Figures 3 and 4). Beginning with the correlated 3-factor model, factor loadings suggest ACE-X task performance is associated with the 3 factors of set reconfiguration, attentional control, and interference resolution. For each of these 3 factors, the strongest indicator for set reconfiguration was TaskSwitch, while the strongest indicators for attentional control and interference resolution were CPT—sustained and Boxed, respectively. Moreover, factor correlations suggested moderate to strong relations between the 3 constructs. The most closely aligned constructs were set reconfiguration and interference resolution (*r*=0.84), followed by attentional control and interference resolution (*r*=0.46), and finally the least aligned constructs were set reconfiguration and attentional control (*r*=0.37). The bifactor model, on the other hand, converged with warnings of a negative variance. Upon inspection of results, a negative residual variance associated with the CPT—impulsive task was the source of the warning. Negative variance estimates can occur for many possible reasons, including outliers [55], underidentification [56,57], model misspecification [56,58-60], and sampling fluctuations [56,61,62]. In the current case, we can reasonably assume that this is caused by a model misspecification; specifically, the attentional control factor is likely underidentified due to only 2 associated indications (CPT-sustained and CPT-impulsive). Because the bifactor model requires estimates of 2 factor loadings per indicator, the model likely fails because we cannot estimate so many parameters with so little information provided. Therefore, we reason that the bifactor model is likely too complex a representation for this set of cognitive data and that associated estimates should be considered unstable. Due to this likely instability of factor loadings associated with these results, we do not interpret factor loadings for the bifactor model here.

**Table 4:** Standardized factor loadings for correlated 3-factor and bifactor models of executive function.

Task	Correlated 3-factor				Bifactor^a^			
	SR^b^	AC^c^	IR^d^	EF^e^		SR	AC	IR
Forward spatial span	0.515	—^f^	—	0.435		0.391	—	—
Backwards spatial span	0.455	—	—	0.341		0.629	—	—
Flanker	—	—	0.56	0.515		—	—	–0.444
Continuous performance (impulsive)	—	0.782	—	0.161		—	1.014	—
Continuous performance (sustained)	—	0.798	—	0.176		—	0.841	—
Stroop	—	—	0.714	0.778		—	—	0.114
Boxed	—	—	0.829	0.809		—	—	–0.151
Compass	—	—	0.641	0.593		—	—	–0.284
TaskSwitch	0.678	—	—	0.603		0.117	—	—
Tap and trace	—	—	0.525	0.518		—	—	–0.046

^a^Model converged with warnings (negative variance).

^b^SR: set reconfiguration.

^c^AC: attentional control.

^d^IR: interference resolution.

^e^EF: executive function.

^f^Not available.

Finally, measures of fit for the network, correlated 3-factor, and bifactor models of EF indicate that the network model provided the best explanation of ACE-X task data (Table 5). The network model provided the best fit to the data according to all measures of fit considered, suggesting that ACE-X task performance can be well described as an interconnected network of EF abilities. Therefore, in the next set of analyses, the network model was used when considering the invariance of parameters across younger and older participants.

**Table 5:** Fit indices for network, correlated 3-factor, and bifactor models of executive function.

Fit index	Network	Correlated 3-factor	Bifactor^a^
Chi-square (*df*)	40.1 (28)	117.03 (32)	345.9 (30)
*P* value	.06	<.001	<.001
CFI^b^	0.99	0.93	0.73
RMSEA^c^ (low-high)	0.029 (0.000-0.047)	0.071 (0.057-0.085)	0.141 (0.128-0.154)
BIC^d^	5075.87	5127.68	5369.07
AIC^e^	4917.71	4986.61	5257.97

^a^Model converged with warnings (negative variance).

^b^CFI: comparative fit index.

^c^RMSEA: root mean square error of approximation.

^d^BIC: Bayesian information criterion.

^e^AIC: Akaike information criterion.

#### Invariance

We next considered the invariance of the network model of EF and found evidence that edge weights for younger and older adults can be considered equivalent (Table 6). We began by fitting the unconstrained model, followed by a model where the edge weights were constrained to be equal, a model where means and edge weights were constrained, and a model where all model parameters were constrained. We first found that the configural model for younger and older participants fit the data well (*χ*^2^_67_=122.7; *P*<.001; CFI=0.95; RMSEA=0.056, 0.040-0.071; BIC=5135.38; AIC=4866.074). However, examining the results of the *χ*^2^ likelihood ratio tests suggests that constraining any of the 3 parameters (edge weights, means, or scaling) resulted in significant detriments to model fit, indicating that younger and older participants differ in their structural organizations of EF. However, when considering information criteria fit indices, which also take model complexity into account, the best-fitting model was the one where edge weights were constrained to equality between younger and older adults. This would suggest that while there may be differences in terms of means and variances between younger and older adults (Table S13 in Multimedia Appendix 1), partial associations among ACE-X task performances can be considered consistent across participants.

**Table 6:** Network invariance fit indices for younger versus older participants.

Fit index	Unconstrained model	Equal edge weights	Equal edge weights+equal means	Equal edge weights+equal means+equal scaling
Chi-square (*df*)	122.67 (67)	102.08 (74)	192.65 (84)	267.78 (96)
Δchi-square (Δ*df*)	—^a^	20.58 (7)	90.57 (10)	75.13 (12)
*P* value	—	.004	<.001	<.001
AIC^b^	4866.07	4831.49	4902.05	4953.18
ΔAIC	—	34.58	70.56	51.13
BIC^c^	5135.38	5070.87	5098.69	5098.52
ΔBIC	—	64.51	27.82	0.17

^a^Not available.

^b^AIC: Akaike information criterion.

^c^BIC: Bayesian information criterion.

#### Relations to Other Variables

Finally, Pearson correlation coefficients between ACE-X and Inquisit tasks suggest mixed evidence in support of concurrent validity for ACE-X tasks (Table 7). Most ACE-X tasks (forward spatial span, Flanker, CPT, Stroop, Boxed, Compass, and TaskSwitch) were only moderately related to the respective Inquisit task. Somewhat unexpectedly, Tap and Trace of ACE-X and the Trail Making task of Inquisit showed the highest degree of task overlap (Table S1 in Multimedia Appendix 1), as these 2 tasks were one of the more divergent task sets. Two sets of tasks, backwards spatial span with backwards Corsi block and Basic Response Time with Simple Visual Response Time, revealed unexpected null associations. Technical issues with the Inquisit backwards Corsi block task could be to blame for the null correlation with its ACE-X counterpart, as 18.9% (25/132) of participants reported to the research team that they were unable to complete this task. This issue also explains the low sample size for this pair of tasks. While technical issues could be responsible for the observed null association between backwards spatial span and the backwards Corsi block task, the low correlation between Basic Response Time and Simple Visual Response Time could potentially be due to various factors, including global platform differences between ACE-X and Inquisit, differences in the handling of dominant versus nondominant hand responding, or elapsed time between trials (refer to subsequent sections for further discussion).

**Table 7:** Pearson correlations between Adaptive Cognitive Evaluation-Explorer and Inquisit tasks.

Task pair	*r*	Values, n (%)	*P* value
Basic response time–simple visual response time	0.26	58 (43.9)	.05
Forward spatial span–forward Corsi block	0.42	67 (50.8)	<.001
Backwards spatial span–backwards Corsi block	−0.05	42 (31.8)	.76
Arrow flanker–letter flanker	0.42	63 (47.7)	.001
CPT^a^ impulsive–TOVA^b^ frequent	0.33	59 (44.7)	.01
CPT sustained–TOVA infrequent	0.39	58 (43.9)	.003
Color-word Stroop–classic Stroop	0.49	74 (56.1)	<.001
Boxed–visual search	0.46	61 (46.2)	<.001
Compass–Posner cueing	0.44	60 (45.5)	<.001
TaskSwitch–category switch	0.31	69 (52.3)	.01
Tap and trace–trail making	0.62	55 (41.7)	<.001

^a^CPT: continuous performance task.

^b^TOVA: tests of variables of attention.

## Discussion

### Principal Findings

As EFs have been shown to predict positive life outcomes and academic success, understanding how to measure them efficiently and accurately is exceedingly important to the field. Here, we presented evidence supporting the reliability and validity of an adaptive, mobile measure of EFs using a large, diverse sample. As previously demonstrated, the incorporation of gamification elements can enhance the sensitivity of a given testing experience through greater participant engagement [32]. While the impact of such gamification directly on task performance and EF measurement has been debated [19], the ability to raise engagement, especially in populations with known elevated performance variability, is a valuable approach to increase testing sensitivity. For adults, ACE-X tasks showed consistency in performance across repeated administrations, as well as a hypothesized network structure, which supported 3 communities of tasks representing set reconfiguration, attentional control, and interference resolution. Moreover, the associations in EF performance suggested by the network model appeared to be consistent across younger and older adults, indicating that the internal structure of ACE-X remained invariant across different ages. Evidence of consistency of performance metrics suggested validity was also supported by comparisons to similar values reported in the literature for both adults and children or adolescents, with future research needed to close the gap in validity evidence for children or adolescents related to test-retest consistency and consistency in internal structures during these developmental periods. While we found strong evidence to support reliability, consistency of performance metrics, and internal structures of ACE-X, evidence related to concurrent validity of ACE-X task performance was mixed. In the subsequent sections, we describe implications of these findings and address concomitant limitations in greater detail.

### Correspondence Between ACE-X Tasks and EF Constructs

Here we found evidence for 3 EF constructs, which we labeled set reconfiguration, attentional control, and interference resolution. While researchers largely agree that EFs can be organized into 3 core constructs, these typically are thought to correspond to inhibitory control, working memory, and cognitive flexibility (the study by Diamond [1] presents a review on EFs). While the correlated 3-factor model was not ultimately selected as the best-fitting model, it does provide some insights into how well these 3 theoretical EF constructs were measured. Examinations of factor loadings suggest that constructs of working memory and inhibitory control were well measured, as indexed by strong associations with the respective EF constructs. Specifically, tasks of forward and backwards spatial span, which are typically used to index aspects of working memory, both showed factor loadings of >0.40, while all other tasks showed strong associations with aspects of inhibitory control constructs with all loadings >0.50 (except for TaskSwitch, which was strongly associated with working memory). While these observations are gathered from the correlated 3-factor model, evidence suggests that the network model fits the data better. More specifically, the network model may provide a better representation of EF task performances as an interconnected network. In such a network, task performance would be directly related to other task performances, as opposed to correlations between task performances and associated EF constructs (as suggested by the correlated 3-factor model). Indeed, examinations of internal structures with a prior iteration of ACE-X, ACE-C, suggest that not only does the network structure well describe associations between task performances but also aligns with developmental theories supporting differentiation of EF constructs over time [13].

On the basis of the results of the network analysis of EFs, the constructs measured by ACE-X do indeed reflect cognitive processes associated with working memory and aspects of inhibitory control, with the engaged cognitive flexibility processes calling upon each of these 2 dominant constructs. The involvement of working memory and inhibitory control in cognitive flexibility abilities has been well documented [63-70], with the present findings replicating such work. For example, forward and backwards spatial span revealed a specific relation with the TaskSwitch paradigm, one of ACE-X’s measures of set reconfiguration. This result is aligned with the literature [63,64,68-70], as working memory is required to recall the cued mappings and enable the constant reconfiguration of a given informational set to successfully complete the TaskSwitch paradigm. A similar pattern emerged regarding the ACE-X tasks indexing interference resolution and the Tap and Trace cognitive flexibility measure [65-67]. Unlike TaskSwitch, the Tap and Trace task design intentionally has an aspect of interference embedded in the task (eg, a visuomotor tracking task while trying to perform the perceptual discrimination task) in addition to the engagement of attentional control through the CPT aspects of the task. Thus, these task design features potentially explain the reason that Tap and Trace was predominantly associated with the interference resolution community, unlike TaskSwitch. Such interpretations are supported by inspection of the network graph: TaskSwitch is centrally located among other tasks but shares relatively weak connections, while Tap and Trace lies on the outskirts of the graph, sharing connections within interference resolution measures.

Using such a mutualistic network model of EFs to examine correspondence between younger and older adults, we found evidence that each set of associations between EF task performances can be considered equivalent. While results of *χ*^2^ likelihood ratio tests suggested no invariant parameters, when considering comparative fit indices, which account for not only the fit of the model to the data but also the complexity of the model, the best-fitting model included equivalent edge weights with all other parameters free to vary. This suggests that for younger and older adults, the association between each set of EF task performances is fundamentally equivalent, but there are differences in the mean and scaling structures of the network model of EFs. This noninvariance of means and scaling is interesting, although perhaps not surprising, as EF has been shown to evolve across the lifespan [71]. Inspection of the unconstrained results for means and scaling suggests that younger adults’ performance levels were better than older adults and that younger adults also tended to be more variable than older adults on average. This is in line with research suggesting that EFs of inhibitory control and working memory capacity tend to follow a U-shaped pattern, where EF performance tends to improve across adolescence into early adulthood, with an eventual leveling and gradual decline across middle and old age.

Consistent with a growing body of literature [10,11,13], our findings suggest support for a hierarchical or mutualistic structure of EF task performance. While hierarchical models express lower-level EF constructs (ie, working memory, inhibition, and cognitive flexibility) as organized under a unifying common EF factor [72,73], mutualistic models (such as the network model here) instead account for, or partial out, what is common among EF task performance to model unique associations (eg, by using partial correlations as presented in the study by Younger et al [13]). Alternatively, in a seminal paper investigating EF development, Friedman et al [10] introduced a nested factor model of EFs, where all tasks instead load onto a common EF factor (inhibition), while updating- and switching-specific tasks were allowed to separately load onto unique factors. This model emphasizes the connected nature of EF tasks while also acknowledging specific task contributions beyond the unifying common EF factor. While the bifactor model examined here is the most similar to this model, we encountered problems in the estimation process, suggesting that this model may be too complex a representation for the EF task performances captured by ACE-X. Instead, this and recent work by our group that examined EF development and network connectedness across middle childhood [13] supports the mutualistic network model, demonstrating performance on the ACE-C software as an interconnected network of EF components. Thus, the present findings provide further support for this growing body of evidence for a hierarchical or mutualistic model of EF task performance, rather than EFs being separate but related constructs. As demonstrated here, the network model performed better than either the correlated 3-factor model or the bifactor model, indicating that a mutualistic structure may be ideal to describe EF task performances.

While agreement on the most appropriate factor structure of EF has not been unanimous, what is clear is that EF task performances share a great deal of communality not fully explained by lower-level constructs. In other words, successful performance on EF tasks will almost always engage more than 1 EF. For example, to hold and manipulate information in mind (working memory), you must first attend to the relevant information while avoiding interference from distractions (interference resolution). Moreover, shifting from one cognitive demand to another (cognitive flexibility) requires updating information related to the new demand (working memory) while suppressing information related to the prior demand (inhibitory control). Therefore, it is imperative that this communality is considered, whether by directly modeling it (eg, in a hierarchical factor structure) or by controlling for it (eg, by using partial correlations in a network model). Here, we have found supporting evidence that the underlying structure of ACE-X follows this same pattern, with a network model that controls for the shared commonality of other EF tasks providing the best fit to the data. Moreover, this network structure remains invariant across younger and older adults, suggesting consistency in the measurement of these EFs.

### Assessing Concurrent Validity Between ACE-X and Similar Tasks

Comparisons between ACE-X and Inquisit revealed mostly moderate and even some null associations within the task sets. There are several reasons for these lower associations between instruments that do not necessarily undermine the validity of ACE-X efforts. First, although similar in design, some task differences may have influenced the ability to detect concurrent validity. For example, Inquisit uses the traditional Corsi-esque approach on the spatial span tasks involving 2 consecutive successful trials to advance and 2 consecutive misses to end a given testing session. Alternatively, ACE-X uses a “3 correct rule” in place for advancement and allows participants to miss 1 trial and still advance so long as the total number of correct responses sums to 3. As another example, while ACE-X’s Basic Response Time task separately measures responding for dominant and nondominant hands, the Simple Visual Reaction Time task of Inquisit does not explicitly indicate which hand the participant should use while responding. A more global platform difference is that Inquisit tasks tend to include more trials and more conditions and take longer to complete than ACE-X tasks, leading to potential participant testing fatigue when compared to ACE-X. Again, using Basic Response Time and Simple Visual Reaction Time as an example, while the number of trials is similar between ACE-X and Inquisit tasks, the time between trials is significantly longer in Inquisit (ranging between 2000 and 8000 ms vs between 800 and 1200 ms for ACE-X). Second, ACE-X tasks were specifically designed to adaptively challenge participants to respond as quickly and accurately as possible, with task difficulty changing on a trial-to-trial basis, unlike Inquisit tasks. The incorporation of this adaptivity, along with the gamified elements, likely made for a very different testing experience between the 2 platforms, and previous work has demonstrated that nonadaptive, nongamified assessments can lead to greater measurement variability [30]. Thus, trying to establish concurrent validity with any other instrument that did not use underlying adaptive mechanics (including Inquisit) was going to lead to a very different testing experience. The utility of ACE-X to assess EFs in a meaningful fashion is demonstrated through the other data presented here, which is warranted given that concurrent validity is often regarded as a weak type of validity if presented on its own [74,75].

Despite achieving mostly weak to moderate associations with Inquisit task performances in our analyses of concurrent validity, the accuracy of ACE-X tasks in measuring EFs is indeed supported by the results of the network analysis. The network model and community detection results presented here garner support for the accuracy of ACE-X as task performances grouped together in specific, predictable clusters consistent with theoretical organizations of EFs. Hypothetically, the use of partial correlations within the network framework helps to account for common method variance, as network edge weights represent commonality between pairs of tasks after variance associated with the full set of ACE-X tasks has been accounted for. What remains is the association specific to that set of task performances, beyond what is shared with the full set of tasks. It is unknown whether and how the task performances on Inquisit measures would form similarly expected clusters. Here, we have chronicled our efforts in understanding the various facets of the validity of ACE-X, although further research is needed to determine which of the sets of measures produce an internal structure that is better aligned with theoretical organizations of EF.

### Limitations and Future Directions

Several limitations should be considered with the present findings. Related to selection of metrics of interest, for many tasks (Flanker, Stroop, Boxed, Compass, and TaskSwitch) we have reported reliability and validity evidence for RCS, rather than some of the more commonly reported metrics for these tasks (eg, accuracy, mean response time, and cost score). We made this decision based on several considerations. First, we felt it important to report validity evidence for a metric that incorporates both accuracy and response time since both are important to a complete understanding of task performance for the indicated tasks. Furthermore, the precedence for the use of this metric has been established with ACE-X’s precursor, ACE-C, as RCS performed well in terms of reliability and as a metric in analyses of internal structure in a large sample of children or adolescents [13,18]. Cost scores, which attempt to capture discrepancies in performance associated with moving from an easier to harder task condition, also present their own set of challenges as they have shown questionable reliability in previous examinations [76]. Here, we have taken a balanced approach by reporting mean response times and accuracy in the consistency of performance metrics section to contrast these commonly reported metrics within the literature but used the RCS metric in other reliability and validity analyses. Because RCS performed well in terms of both reliability and validity for all tasks where we used this metric, we found it unnecessary to explore the validity of other possible metrics here.

Moreover, some of our statistical comparisons were limited by the task metric of interest when it came to forward and backwards spatial span tasks. Because these tasks are limited by the number of items that can be stored in short-term memory, we are limited to a standard range of about 7 (SD 2). This restricted range results in limited variability, making it more difficult to find significant relations. This restricted range also creates a problem for test-retest correlations, as even relatively minor improvements in task performance become exaggerated by the narrow possible range of scores. While we acknowledge this as a limitation here, it does not necessarily reflect a problem with these tasks or their validity, though other metrics that consider the number of trials needed to get to the final object span may be better equipped to handle subtle variations in task performance by capturing a more continuous measure of performance and should be considered in the future.

One relevant concern of this work involves our reliance on MTurk and other social media platforms in recruiting participants. Consequently, aspects related to the representativeness of the said participants should be considered when evaluating the generalizability of the findings. Previous work described the lack of heterogeneity in users that can arise from such recruitment strategies, which can in turn foster a bias in collected data and subsequent interpretations. Alternatively, others have argued that such drawbacks do not warrant precluding the use of said platforms [77], as recruiting biases are often present in most research studies [78,79]. In either case, the present findings should be considered with both perspectives in mind.

While ACE-X was designed to measure EFs for individuals aged ≥7 years, the addition of specific design facets may facilitate the use of ACE-X in even younger populations. For example, the incorporation of in-app tutorials that guide new users through the user interface and key features can be beneficial in demonstrating how to use a given feature even with limited literacy. Similarly, there are many games whose instructional design allows users to explore, without consequences, how to play a given game, especially for children aged <7 years [80]. These types of mechanics warrant further exploration not only for potentially enhancing the reach of ACE-X but also for other digital tools that could benefit a younger population.

It is true that digital health technologies, such as ACE-X, can provide a potentially direct, cost-efficient, and convenient way to allow researchers to gather data in a multitude of settings. However, it should be noted that the effectiveness of EF assessments can vary significantly across different cultural contexts [81]. Future studies in this regard with ACE-X are warranted, as rigorous studies of cultural adaptation in the digital context are noted as being scarce [82], and the ACE-X software architecture allows for the ability to easily change aspects, including avatars and language.

Finally, a significant limitation here was the lack of validity evidence collected for child or adolescent participants. Changes brought about by the COVID-19 pandemic left us with limited access to schools and, therefore, limited control in terms of task administration and timing. We were often limited to administering only 2 or 3 possible task orders with a very narrow window of time for task administration. This meant that often we were only able to collect task data for a specific predetermined construct set. While we have made efforts to overcome challenges brought about by the COVID-19 pandemic and have acknowledged specific known impacts, it is always possible that some other unknown influence may have affected the reliability and validity of the results presented here. Because of these limitations, while we did find support for reasonable performance metrics contrasted to similar tasks in the literature, future research will be needed to adequately assess other evidence of reliability and validity for children and adolescents, perhaps making use of planned missingness designs to keep study demands reasonable for these young participants.

### Conclusions

The evidence presented here supports the use of ACE-X as a measure for understanding individual differences in EFs as well as those EFs’ relations to external variables. The possibility of using ACE-X performance as a diagnostic or screening tool for understanding potential deficits in EF performance is still to be determined. This would require careful selection of normative and clinical samples of test takers to create comparison groups and establish appropriate ranges for cut points.

The COVID-19 pandemic provided the initial push toward ecological validity through assessing EFs in familiar home environments and increased the need for valid and reliable remote research tools. Here, with >6000 participants, we have provided evidence in support of ACE-X, a mobile, adaptive set of cognitive tasks with engaging language and immersive graphics, as a reliable and valid measure of EFs. We expect that as technology continues to progress, ACE-X and other similar gamified versions of cognitive tasks will become integral in understanding relational patterns between EFs and important life outcomes. While we have made significant headway in uncovering evidence in support of reliability and validity for adult participants, there is still work to be done to recreate these findings with children and adolescents. However, these validation efforts provide evidence that this gamified research tool could play a pivotal role in the world of remote data collection while advancing the methods used to assess EFs in real-world settings.

## Appendix

Multimedia Appendix 1 Supplementary text, tables, and figures for the validation of Adaptive Cognitive Evaluation-Explorer.

## Abbreviations

ACE-C: Adaptive Cognitive Evaluation-Classroom
ACE-X: Adaptive Cognitive Evaluation-Explorer
ADHD: attention-deficit/hyperactivity disorder
AIC: Akaike information criterion
BIC: Bayesian information criterion
CFI: comparative fit index
CPT: continuous performance task
EF: executive function
ICC: intraclass correlation coefficient
MTurk: Amazon Mechanical Turk
RCS: rate correct score
RMSEA: root mean square error of approximation
TOVA: test of variables of attention

## Data Availability

The datasets generated or analyzed during this study, as well as the Adaptive Cognitive Evaluation-Explorer software, are available from the corresponding author upon reasonable request.
