# Supplementary material for: Validation of an Adaptive Assessment of Executive Functions (Adaptive Cognitive Evaluation-Explorer): Longitudinal and Cross-Sectional Analyses of Cognitive Task Performance
Source: J Med Internet Res. 2025 Apr 21;27:e60041. doi: 10.2196/60041 (PMC12053272; doi:10.2196/60041)
Supplement: Multimedia Appendix 1 [file jmir_v27i1e60041_app1.docx]

# Multimedia Appendix

This is a Multimedia Appendix to a full manuscript published in the J Med Internet Res. For full copyright and citation information see http://dx.doi.org/10.2196/jmir.60041

### Subject Removal Criteria

For all adult data collected remotely as part of the Adaptive Cognitive Evaluation-Explorer (ACE-X) Validation Sample, we took a multi-tiered approach to filtering data from ‘bad’ and ‘good’ actors. First, we automatically labeled respondents who demonstrated any high-risk flags (completion outside of the US, use of disposable email, duplicated IP address) as ‘bad actors’ (see Table S15 below for full list and descriptions of high/moderate risk flags). This automated review process resulted in 2,888 respondents (75% of adult respondents) being labeled as bad actors. Next, for participants with no high-risk flags, we labeled participants with a high number of moderate-risk flags (4 or more flags) as bad actors; this resulted in an additional 56 respondents (1% of adult respondents) being labeled as bad actors.

Despite these efforts to remove data from bad actors using the aforementioned process, there were still some clearly identifiable bad actors that managed to slip past our automated detections with no flags for inconsistent responding. Therefore, all remaining participants were labeled as “low-risk”, but subjected to an internal review process. First, a list of low-risk participant identification numbers was created. Next, two researchers independently evaluated the apparent authenticity of each record. This review included inspection of the record in question as well as adjacent responses to the eligibility survey. Each record was inspected for repetitive or patterned responding, inconsistent responding, and reporting of false information (e.g., identifying themselves by the names of celebrities or fictional characters). If after independent review the two researchers failed to reach agreement about a particular respondent, a third researcher acted as tiebreaker as to the authenticity of the record. After establishing substantial interrater agreement during initial review (Cohen’s kappa = .81; 95% CI [.77; .85]), we removed the requirement of independent review by two researchers, and instead, a single researcher reviewed all relevant responses and only those that were rated with low confidence were reviewed by a second researcher (and a third in the event of a tie). This process resulted in an additional 273 respondents (7% of adult respondents) being labeled as bad actors after review. The remaining 606 adult participants (16% of adult respondents) were considered good actors in further analyses.

### Supplementary Figure


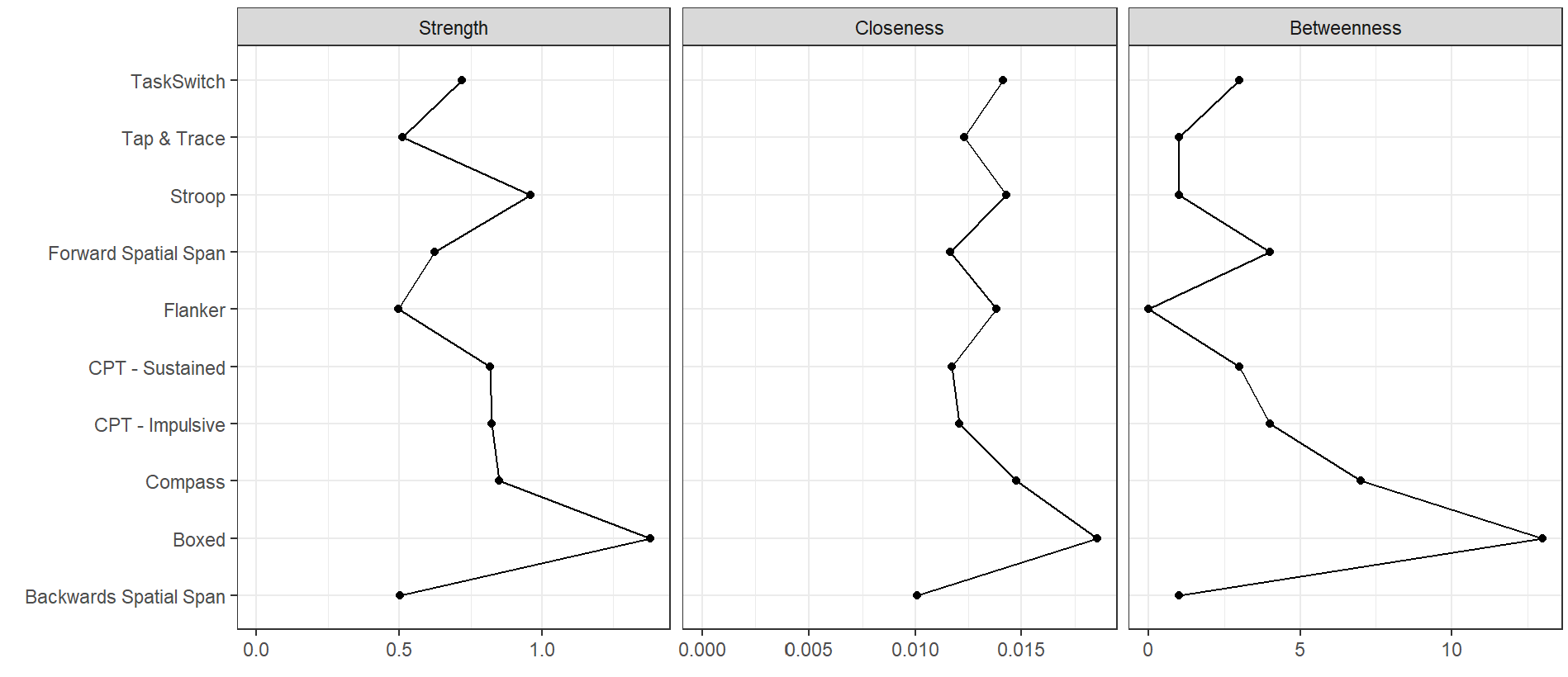


**Figure S1.** Measures of centrality from network model of ACE-X task performance. CPT = continuous performance task.

### Supplementary Tables

Table S1. ACE-X and Inquisit™ task comparisons.

| **Domain** | **ACE-X Task** | **Analog Inquisit Task** | **Task Similarities** | **Differences from Analog ACE-X Task** |
| --- | --- | --- | --- | --- |
| **Processing Speed** | *Basic Response Time* | *Simple Visual Reaction Time Task* | - Participant presses key as soon as target stimulus appears - Stimuli always appear in center of screen | - Does not separate dominant/non-dominant hand trials - Not adaptive |
| **Working Memory** | *Forward/Backwards Spatial Span* (Corsi, 1972) | *Forward/Backwards Corsi Block* (Corsi, 1972; Kessels et al., 2000) | - 9 possible spatial locations - Sequence identified by boxes/diamonds “lighting-up” in pre-defined order - Sequence length increases after correct sequence recall | - Sequence length ranges from 2 to 8 objects - Requires only 1 correctly recalled sequence to advance - Allows only 2 chances to recall each sequence |
| **Inhibitory Control** | *Arrow Flanker* (Eriksen & Eriksen, 1974) | *Letter Flanker Task* (Eriksen & Eriksen, 1974) | - Target flanked by 0 or 4 objects - Target object is center object | - Stimuli appear as letters - Difficulty controlled by number of distractors and similarity with target letter - Not adaptive |
|  | *Continuous Performance Task* (Greenberg et al., 1991) | *Test of Variables of Attention* (Greenberg & Waldman, 1993; Greenberg et al., 2016) | - Not adaptive - Measures of impulsive and sustained attention - Targets appear at top of screen - Nontargets appear at bottom of screen | - ~22.2% of sustained trials are targets - ~77.8% of impulsive trials are targets |
|  | *Color-word Stroop* (Stroop, 1935; Mead et al., 2002) | *Classic Stroop Task* (Stroop; 1935) | - Participants attend to the color of the word and ignore the color the word spells - Include neutral/congruent/incongruent trials | - Not adaptive |
|  | *Boxed* (Treisman & Gelade,1980) | *Visual Search Task* (Motter & Simoni, 2008) | - Include conjunction trials - Number of targets and distractors varies | - Stimuli appear as letters - Target letter shares one feature with each distractor letter - No feature-only trials - Not adaptive |
|  | *Compass* (Posner et al., 1980) | *Posner Cueing Task* (Posner et al., 1980) | - Target appears in one of two triangles/boxes - Include neutral/valid/invalid trials - Include arrow conditions | - Also includes “highlight” conditions, where box is briefly highlighted before target appears - Not adaptive |
| **Cognitive Flexibility** | *TaskSwitch* (see Monsell, 2003 for review) | *Category Switch Task* (Friedman et al., 2008; Mayr & Kleigl, 2000) | - Participants tasked with switching between attending to 2 target features | - Participants tasked with switching between attending to “living” aspect (living vs. non-living object) and “size” aspect (bigger vs. smaller than a basketball) - Not adaptive |
|  | *Tap & Trace* (Eversheim & Bock, 2001) | *Trail Making Task* (Armitages, 1946; Reitan, 1958) | - Participants trace a path while performing the instructed behavior | - In dual condition, participants trace a path between alphanumeric characters, alternating between letters and numbers - Not adaptive |

Table S2. Selected comparison literature with metric ranges for ACE-X response process validation.

| **ACE-X Task** | **Reference** | **Age Range(s)** | **Metric(s)** | **Min** | **Max** |
| --- | --- | --- | --- | --- | --- |
| **Basic Response Time** | Anguera et al. (2023) [S1] | 8-12 | RT^a^ | 357 ms | 357 ms |
|  | Anguera et al. (2022) [S2] | 60-85 | RT | 409 ms | 409 ms |
|  | Anguera et al. (2017) [S3] | 8-12 | RT | 418 ms | 418 ms |
|  | Anguera et al. (2013) [S4] | 60-80 | RT | 297 ms | 297 ms |
|  | Bleecker et al. (1987) [S5] | 40-89 | RT | 231 ms | 310 ms |
|  | Eckner et al. (2010) [S6] | 18-23 | RT | 268 ms | 268 ms |
|  | Gallen et al. (2021) [S7] | 8-12 | RT | 518 ms | 518 ms |
|  | Lemay et al. (2004) [S8] | 52-80 | RT | 276 ms | 276 ms |
|  | Rolle et al. (2017) [S9] | 18-85 | RT | 330 ms | 359 ms |
|  | Silverman (2010) [S10] | 16-36 | RT | 183 ms | 324 ms |
|  | Stuss et al. (1989) [S11] | 20-69 | RT | 232 ms | 250 ms |
|  | Zanto & Gazzaley (2013) [S12] | 18-80 | RT | 302 ms | 353 ms |
|  | Zanto et al. (2011) [S13] | 18-80 | RT | 305 ms | 350 ms |
|  | Ziegler et al. (2019) [S14] | 60-85 | RT | 332 ms | 332 ms |
| **Stroop** | Banich et al. (2000) [S15] | 20-35 | Incongruent RT | 818 ms | 818 ms |
|  |  |  | Neutral RT | 679 ms | 679 ms |
|  | Fan et al. (2003) [S16] | 18-29 | Congruent Error Rate | 2.86% | 2.86% |
|  |  |  | Incongruent Error Rate | 5.26% | 5.26% |
|  |  |  | Neutral Error Rate | 3.00% | 3.00% |
|  |  |  | Congruent RT | 650 ms | 650 ms |
|  |  |  | Incongruent RT | 790 ms | 790 ms |
|  |  |  | Neutral RT | 696 ms | 696ms |
|  | Hasshim et al. (2019) [S17] | 18-29 | Congruent RT | 594 ms | 594 ms |
|  |  |  | Incongruent RT | 627 ms | 627 ms |
|  | Heathcote et al. (1991) [S18] | 18-29 | Congruent RT | 605 ms | 605 ms |
|  |  |  | Incongruent RT | 700 ms | 700 ms |
|  | Mead et al. (2002) [S19] | 18-46 | Congruent Accuracy | 97.6% | 97.6% |
|  |  |  | Incongruent Accuracy | 95.3% | 95.3% |
|  |  |  | Neutral Accuracy | 96.6% | 96.6% |
|  |  |  | Congruent RT | 673 ms | 673 ms |
|  |  |  | Incongruent RT | 781 ms | 781 ms |
|  |  |  | Neutral RT | 725 ms | 725 ms |
|  | Milham et al. (2001) [S20] | 18-30 | Incongruent RT | 701 ms | 701 ms |
|  |  |  | Neutral RT | 658 ms | 658 ms |
|  | Toth et al. (2019) [S21] | 17-34 | Congruent RT | 730 ms | 730 ms |
|  |  |  | Incongruent RT | 814 ms | 814 ms |
| **Flanker** | Eriksen & Eriksen (1974) [S22] | 18-29 | Congruent RT | 440 ms | 440 ms |
|  |  |  | Incongruent RT | 470 ms | 470 ms |
|  | Fan et al. (2003) [S16] | 18-34 | Congruent Error Rate | 0.5% | 0.5% |
|  |  |  | Incongruent Error Rate | 4.0% | 4.0% |
|  |  |  | Neutral Error Rate | 1.0% | 1.0% |
|  |  |  | Congruent RT | 475 ms | 475 ms |
|  |  |  | Incongruent RT | 559 ms | 559 ms |
|  |  |  | Neutral RT | 470 ms | 470 ms |
|  | Haciahmet et al. (2021) [S23] | 20-28 | Congruent Accuracy | 97.3% | 97.3% |
|  |  |  | Incongruent Accuracy | 96.3% | 96.3% |
|  |  |  | Congruent RT | 543 ms | 543 ms |
|  |  |  | Incongruent RT | 567 ms | 567 ms |
|  | Markiewicz et al. (2022) [S24] | 18-28 | Congruent RT | 350 ms | 350 ms |
|  |  |  | Incongruent RT | 410 ms | 410 ms |
|  | Servant & Logan (2019) [S25] | 20-30 | Congruent RT | 360 ms | 360 ms |
|  |  |  | Incongruent RT | 420 ms | 420 ms |
|  | Stoffels & van der Molen (1988) [S26] | 20-25 | Congruent RT | 450 ms | 450 ms |
|  |  |  | Incongruent RT | 510 ms | 510 ms |
|  | Wang et al. (2023) [S27] | 18-32 | Congruent Accuracy | 99.3% | 99.3% |
|  |  |  | Incongruent Accuracy | 88.9% | 88.9% |
|  |  |  | Congruent RT | 448 ms | 448 ms |
|  |  |  | Incongruent RT | 569 ms | 569 ms |
| **TaskSwitch** | Dykstra et al. (2022) [S28] | 18-29 | Switch RT | 810 ms | 810 ms |
|  |  |  | Stay RT | 790 ms | 790 ms |
|  | Erb, Touron & Marcovitch (2020) [S29] | 18-75 | Error Rate | 0.07% | 0.08% |
|  |  |  | Switch RT | 830 ms | 960 ms |
|  |  |  | Stay RT | 775 ms | 875 ms |
|  | Koch et al. (2023) [S30] | 18-35 | Switch Accuracy | 93% | 93% |
|  |  |  | Stay Accuracy | 97% | 97% |
|  |  |  | Switch RT | 550 ms | 550 ms |
|  |  |  | Stay RT | 500 ms | 500 ms |
|  | Kray & Lindenberger (2000) [S31] | 20-80 | Switch RT | 730 ms | 730 ms |
|  |  |  | Stay RT | 675 ms | 675 ms |
|  | Mayr & Kliegl (2003) [S32] | 18-29 | Switch RT | 1440 ms | 1440 ms |
|  |  |  | Stay RT | 1020 ms | 1020 ms |
|  | Monsell (2003) [S33] | 19-38 | Switch RT | 750 ms | 750 ms |
|  |  |  | Stay RT | 610 ms | 610 ms |
|  | Rogers & Monsell (1995) [S34] | 21-46 | Error Rate | 7.6% | 7.6% |
|  |  |  | Switch RT | 888 ms | 888 ms |
|  |  |  | Stay RT | 679 ms | 679 ms |
| **Visual Search (*Boxed*)** | Ajana et al. (2023) [S35] | 21-83 | Feature RT | 1750 ms | 1750 ms |
|  |  |  | Conjunction RT | 2250 ms | 2250 ms |
|  | Becker (2010) [S36] | 18-29 | Feature RT | 1218 ms | 1218 ms |
|  |  |  | Conjunction RT | 1882 ms | 1882 ms |
|  | Manley et al. (2023) [S37] | 18-26 | Feature RT | 888 ms | 888 ms |
|  |  |  | Conjunction RT | 1156 ms | 1156 ms |
|  | Merenstein et al. (2023) [S38] | 18–78 | Feature RT | 690 ms | 690 ms |
|  |  |  | Conjunction RT | 907 ms | 907 ms |
|  | Pennington et al. (2019) [S39] | 18-25 | Feature RT | 1000 ms | 1000 ms |
|  |  |  | Conjunction RT | 1150 ms | 1150 ms |
|  | Plude & Doussard-Roosevelt (1989) [S40] | 17-71 | Feature Accuracy | 98% | 99% |
|  |  |  | Conjunction Accuracy | 89% | 89% |
|  |  |  | Feature RT | 600 ms | 855 ms |
|  |  |  | Conjunction RT | 800 ms | 1220 ms |
|  | Treisman & Gelade (1980) [S41] | 24-29 | Error Rate | 0.8% | 4.9% |
|  |  |  | Feature RT | 446 ms | 446 ms |
|  |  |  | Conjunction RT | 422 ms | 422 ms |
| **Tests of Variables of Attention (*Continuous Performance Task*)** | Anguera et al. (2023) [S1] | 7-12 | Sustained RT | 491 ms | 491 ms |
|  |  |  | Impulsive RT | 451 ms | 451 ms |
|  | Anguera et al. (2017) [S3] | 8-12 | Sustained RT | 450 ms | 450 ms |
|  |  |  | Impulsive RT | 360 ms | 360 ms |
|  | Anguera et al. (2016) [S42] | 60-80 | Sustained RT | 360 ms | 360 ms |
|  |  |  | Impulsive RT | 327 ms | 327 ms |
|  | Anguera et al. (2013) [S4] | 60-85 | Sustained RT | 346 ms | 346 ms |
|  |  |  | Impulsive RT | 294 ms | 294 ms |
|  | Greenberg & Waldman (1993) [S43] | 6-15 | Sustained RT | 350 ms | 626 ms |
|  | Memoria et al. (2018) [S44] | 38-68 | Sustained RT | 406 ms | 406 ms |
|  |  |  | Impulsive RT | 300 ms | 460 ms |
|  | Ziegler et al. (2019) [S14] | 18-29 | Sustained RT | 373 ms | 373 ms |
|  |  |  | Impulsive RT | 307 ms | 307 ms |
| **Posner Cueing Task (*Compass*)** | Arif et al. (2020) [S45] | 30-60 | Valid RT | 1007 ms | 1007 ms |
|  |  |  | Invalid RT | 1075 ms | 1075 ms |
|  | Dukewich (2008) [S46] | 18-25 | Valid RT | 455 ms | 455 ms |
|  |  |  | Invalid RT | 485 ms | 485 ms |
|  | Johnson & Haggard (2003) [S47] | 23-51 | Valid RT | 332 ms | 332 ms |
|  |  |  | Invalid RT | 351 ms | 351 ms |
|  | Merritt et al. (2005) [S48] | 18-35 | Valid RT | 240 ms | 290 ms |
|  |  |  | Invalid RT | 275ms | 360 ms |
|  | Perez-Edgar & Fox (2005) [S49] | 7 | Valid RT | 848 ms | 848 ms |
|  |  |  | Invalid RT | 885 ms | 885 ms |
|  | Posner et al. (1980) [S50] | 18-24 | Valid RT | 267 ms | 267 ms |
|  |  |  | Invalid RT | 270 ms | 270 ms |
|  | Van Der Stigchel & Theeuwes (2007) [S51] | 19-32 | Valid RT | 421 ms | 421 ms |
|  |  |  | Invalid RT | 449 ms | 449 ms |
| **Multitasking Task (*Tap and Trace*)** | Anguera et al. (2016) [S42] | 8-12 | Single Task RT | 491 ms | 491 ms |
|  |  |  | Multitask RT | 591 ms | 591 ms |
|  | Anguera et al. (2013) [S4] | 60-80 | Single Task RT | 394 ms | 484 ms |
|  |  |  | Multitask RT | 444 ms | 601 ms |
|  | Bherer et al. (2008) [S52] | 18-76 | Single Task Accuracy | 92% | 95% |
|  |  |  | Multitask Accuracy | 92% | 93% |
|  |  |  | Single Task RT | 450 ms | 900 ms |
|  |  |  | Multitask RT | 900 ms | 1280 ms |
|  | Eversheim & Bock (2001) [S53] | 21-30 | Single Task RT | 360 ms | 360 ms |
|  |  |  | Multitask RT | 450 ms | 450 ms |
| **Forward Spatial Span** | Chen et al. (2009) [S54] | 18-77 | Mean Span Length | 6.2 | 6.2 |
|  | Corsi (1973) [S55] | 28 | Mean Span Length | 4.9 | 4.9 |
|  | Farrell Pagulayan et al. (2006) [S56] | 7-26 | Mean Span Length | 6.2 | 6.4 |
|  | Hazarika & Dasgupta (2018) [S57] | 18-22 | Mean Span Length | 5.0 | 6.2 |
|  | Kessels et al. (2008) [S58] | 50-92 | Mean Span Length | 4.8 | 5.3 |
|  | Mammarela & Cornoldi (2005) [S59] | 8-11 | Mean Span Length | 5.4 | 5.4 |
|  | Siddi et al. (2020) [S60] | 18-65 | Mean Span Length | 7.5 | 9.6 |
|  | Vandierendonck et al. (2004) [S61] | 17-19 | Mean Span Length | 6.0 | 6.0 |
| **Backward Spatial Span** | Chen et al. (2009) [S54] | 18-77 | Mean Span Length | 6.1 | 6.1 |
|  | de Paula et al. (2016) [S62] | 70-84 | Mean Span Length | 2.8 | 2.8 |
|  | Furley & Memmert (2010) [S63] | 18-27 | Mean Span Length | 4.2 | 4.2 |
|  | Hazarika & Dasgupta (2018) [S57] | 18-22 | Mean Span Length | 5.6 | 5.6 |
|  | Siddi et al. (2020) [S60] | 18-65 | Mean Span Length | 6.4 | 9.1 |
|  | Vandierendonck et al. (2004) [S61] | 17-19 | Mean Span Length | 5.6 | 5.6 |
| ^a^RT = response time. | | | | | |

Table S3. ACE-X mean performance metrics across age groups for Basic Response Time.

|  |  | **Basic Response Time** | | | | | |
| --- | --- | --- | --- | --- | --- | --- | --- |
| **Age Group** |  | **Overall** | | **Dominant** | | **Nondominant** | |
|  | **Metric** | **M** | **SD** | **M** | **SD** | **M** | **SD** |
| **12 and under** | *Response Time* | 341.63 | 45.93 | 330.78 | 43.76 | 367.09 | 89.22 |
| **13-17 years** | *Response Time* | 328.62 | 43.40 | 319.34 | 41.83 | 354.05 | 103.04 |
| **18-22 years** | *Response Time* | 301.45 | 40.50 | 294.02 | 38.68 | 326.50 | 83.47 |
| **23-29 years** | *Response Time* | 309.03 | 41.78 | 302.03 | 40.77 | 393.53 | 267.68 |
| **30-39 years** | *Response Time* | 325.39 | 55.48 | 316.85 | 55.46 | 360.33 | 113.35 |
| **40+ years** | *Response Time* | 363.82 | 76.69 | 339.34 | 61.65 | 438.17 | 214.21 |

Table S4. ACE-X mean performance metrics across age groups for Forward and Backward Spatial Span.

|  |  | **Spatial Span** | | | |
| --- | --- | --- | --- | --- | --- |
| **Age Group** |  | **Forward** | | **Backwards** | |
|  | **Metric** | **M** | **SD** | **M** | **SD** |
| **12 and under** | *Response Time* | 3179.46 | 801.02 | 3529.83 | 1021.12 |
|  | *Object Span* | 6.86 | 0.83 | 6.31 | 0.79 |
| **13-17 years** | *Response Time* | 3077.19 | 753.39 | 3211.17 | 953.39 |
|  | *Object Span* | 7.11 | 0.89 | 6.47 | 0.93 |
| **18-22 years** | *Response Time* | 2986.33 | 630.74 | 3142.48 | 1015.09 |
|  | *Object Span* | 7.48 | 0.97 | 6.91 | 1.02 |
| **23-29 years** | *Response Time* | 3289.70 | 844.57 | 3340.25 | 964.78 |
|  | *Object Span* | 7.40 | 0.86 | 6.83 | 0.99 |
| **30-39 years** | *Response Time* | 3295.75 | 958.46 | 3296.59 | 1074.10 |
|  | *Object Span* | 7.32 | 0.92 | 6.71 | 0.99 |
| **40+ years** | *Response Time* | 3631.42 | 947.19 | 3808.48 | 1092.48 |
|  | *Object Span* | 7.03 | 0.83 | 6.53 | 0.86 |

Table S5. ACE-X mean performance metrics across age groups for Flanker.

|  |  | **Flanker** | | | | | |
| --- | --- | --- | --- | --- | --- | --- | --- |
| **Age Group** |  | **Overall** | | **Congruent** | | **Incongruent** | |
|  | **Metric** | **M** | **SD** | **M** | **SD** | **M** | **SD** |
| **12 and under** | *Response Time* | 633.40 | 191.31 | 609.48 | 174.79 | 657.56 | 220.10 |
|  | *Accuracy* | 88% | 10% | 93% | 9% | 82% | 15% |
| **13-17 years** | *Response Time* | 579.81 | 188.89 | 559.05 | 177.24 | 600.46 | 209.24 |
|  | *Accuracy* | 89% | 9% | 94% | 8% | 83% | 15% |
| **18-22 years** | *Response Time* | 480.98 | 244.57 | 460.21 | 208.26 | 501.69 | 285.07 |
|  | *Accuracy* | 90% | 8% | 96% | 8% | 85% | 13% |
| **23-29 years** | *Response Time* | 556.95 | 297.71 | 542.18 | 289.36 | 570.93 | 307.20 |
|  | *Accuracy* | 90% | 7% | 97% | 5% | 84% | 11% |
| **30-39 years** | *Response Time* | 513.12 | 107.07 | 499.82 | 107.34 | 526.14 | 109.73 |
|  | *Accuracy* | 92% | 7% | 98% | 5% | 87% | 12% |
| **40+ years** | *Response Time* | 610.85 | 218.16 | 597.46 | 218.91 | 623.68 | 219.56 |
|  | *Accuracy* | 93% | 7% | 97% | 6% | 89% | 10% |

Table S6. ACE-X mean performance metrics across age groups for the Continuous Performance Task.

|  |  | **Continuous Performance Task** | | | |
| --- | --- | --- | --- | --- | --- |
| **Age Group** |  | **Impulsive** | | **Sustained** | |
|  | **Metric** | **M** | **SD** | **M** | **SD** |
| **12 and under** | *Response Time* | 442.28 | 103.41 | 525.89 | 132.61 |
|  | *Accuracy* | 88% | 10% | 78% | 27% |
| **13-17 years** | *Response Time* | 436.99 | 118.03 | 512.30 | 140.65 |
|  | *Accuracy* | 89% | 10% | 85% | 24% |
| **18-22 years** | *Response Time* | 360.59 | 48.68 | 459.00 | 103.56 |
|  | *Accuracy* | 97% | 5% | 95% | 13% |
| **23-29 years** | *Response Time* | 461.85 | 261.64 | 519.01 | 214.11 |
|  | *Accuracy* | 96% | 8% | 95% | 11% |
| **30-39 years** | *Response Time* | 405.65 | 82.69 | 478.66 | 134.25 |
|  | *Accuracy* | 97% | 4% | 96% | 10% |
| **40+ years** | *Response Time* | 445.84 | 143.27 | 517.62 | 151.69 |
|  | *Accuracy* | 98% | 4% | 98% | 6% |

Table S7. ACE-X mean performance metrics across age groups for Stroop.

|  |  | **Stroop** | | | | | |
| --- | --- | --- | --- | --- | --- | --- | --- |
| **Age Group** |  | **Overall** | | **Congruent** | | **Incongruent** | |
|  | **Metric** | **M** | **SD** | **M** | **SD** | **M** | **SD** |
| **12 and under** | *Response Time* | 810.85 | 266.79 | 773.73 | 237.50 | 848.55 | 305.09 |
|  | *Accuracy* | 91% | 7% | 95% | 7% | 87% | 11% |
| **13-17 years** | *Response Time* | 700.05 | 192.51 | 672.02 | 175.99 | 728.60 | 217.52 |
|  | *Accuracy* | 92% | 7% | 95% | 6% | 89% | 10% |
| **18-22 years** | *Response Time* | 548.63 | 107.12 | 525.53 | 89.23 | 572.26 | 128.73 |
|  | *Accuracy* | 93% | 6% | 95% | 7% | 91% | 7% |
| **23-29 years** | *Response Time* | 674.58 | 354.30 | 654.00 | 345.79 | 695.35 | 364.39 |
|  | *Accuracy* | 92% | 8% | 94% | 8% | 90% | 11% |
| **30-39 years** | *Response Time* | 660.10 | 183.89 | 636.08 | 167.95 | 684.56 | 205.11 |
|  | *Accuracy* | 93% | 6% | 95% | 6% | 92% | 8% |
| **40+ years** | *Response Time* | 832.66 | 297.14 | 796.39 | 277.97 | 869.33 | 322.69 |
|  | *Accuracy* | 94% | 8% | 97% | 5% | 90% | 14% |

Table S8. ACE-X mean performance metrics across age groups for Boxed.

|  |  | **Boxed** | | | | | | | | | |
| --- | --- | --- | --- | --- | --- | --- | --- | --- | --- | --- | --- |
| **Age Group** |  | **Overall** | | **Feature 4** | | **Feature 12** | | **Conjunction 4** | | **Conjunction 12** | |
|  | **Metric** | **M** | **SD** | **M** | **SD** | **M** | **SD** | **M** | **SD** | **M** | **SD** |
| **12 and under** | *Response Time* | 977.53 | 204.46 | 802.35 | 158.80 | 825.53 | 166.20 | 1028.05 | 249.17 | 1284.50 | 367.03 |
|  | *Accuracy* | 86% | 8% | 87% | 9% | 89% | 10% | 86% | 11% | 82% | 14% |
| **13-17 years** | *Response Time* | 883.05 | 163.58 | 729.78 | 149.90 | 739.80 | 136.52 | 924.44 | 198.89 | 1164.67 | 287.66 |
|  | *Accuracy* | 89% | 7% | 90% | 9% | 91% | 7% | 89% | 9% | 86% | 13% |
| **18-22 years** | *Response Time* | 737.17 | 126.90 | 612.91 | 99.93 | 628.43 | 99.83 | 748.96 | 135.02 | 979.84 | 213.53 |
|  | *Accuracy* | 91% | 5% | 92% | 8% | 93% | 6% | 90% | 7% | 91% | 7% |
| **23-29 years** | *Response Time* | 809.39 | 257.62 | 690.61 | 246.41 | 703.79 | 253.92 | 814.56 | 262.50 | 1047.27 | 332.80 |
|  | *Accuracy* | 90% | 7% | 92% | 7% | 91% | 8% | 89% | 9% | 87% | 12% |
| **30-39 years** | *Response Time* | 825.00 | 141.79 | 698.37 | 126.44 | 700.57 | 120.95 | 830.42 | 154.94 | 1098.98 | 224.22 |
|  | *Accuracy* | 92% | 5% | 93% | 7% | 93% | 7% | 91% | 7% | 90% | 9% |
| **40+ years** | *Response Time* | 957.06 | 205.79 | 810.21 | 189.63 | 805.10 | 185.64 | 949.59 | 207.49 | 1298.19 | 308.46 |
|  | *Accuracy* | 92% | 5% | 94% | 6% | 94% | 6% | 91% | 7% | 90% | 10% |

Table S9. ACE-X mean performance metrics across age groups for Compass.

|  |  | **Compass** | | | | | | | |
| --- | --- | --- | --- | --- | --- | --- | --- | --- | --- |
| **Age Group** |  | **Overall** | | **Valid** | | **Invalid** | | **Neutral** | |
|  | **Metric** | **M** | **SD** | **M** | **SD** | **M** | **SD** | **M** | **SD** |
| **12 and under** | *Response Time* | 398.31 | 75.81 | 384.10 | 75.48 | 438.51 | 88.74 | 414.59 | 85.04 |
|  | *Accuracy* | 98% | 2% | 98% | 2% | 95% | 8% | 99% | 4% |
| **13-17 years** | *Response Time* | 375.61 | 70.81 | 363.74 | 70.49 | 407.76 | 76.89 | 390.53 | 80.07 |
|  | *Accuracy* | 98% | 3% | 99% | 3% | 95% | 7% | 99% | 4% |
| **18-22 years** | *Response Time* | 338.85 | 130.11 | 327.80 | 129.33 | 366.86 | 137.19 | 354.48 | 129.73 |
|  | *Accuracy* | 99% | 1% | 100% | 1% | 97% | 7% | 100% | 2% |
| **23-29 years** | *Response Time* | 400.86 | 186.59 | 389.53 | 184.29 | 427.40 | 191.36 | 420.34 | 195.57 |
|  | *Accuracy* | 99% | 2% | 99% | 2% | 96% | 7% | 99% | 3% |
| **30-39 years** | *Response Time* | 377.33 | 93.37 | 368.11 | 92.96 | 399.85 | 97.24 | 391.67 | 98.34 |
|  | *Accuracy* | 99% | 1% | 99% | 2% | 97% | 6% | 100% | 2% |
| **40+ years** | *Response Time* | 470.01 | 436.30 | 454.55 | 397.56 | 514.47 | 588.52 | 489.38 | 459.27 |
|  | *Accuracy* | 99% | 2% | 99% | 2% | 97% | 6% | 99% | 3% |

Table S10. ACE-X mean performance metrics across age groups for TaskSwitch.

|  |  | **TaskSwitch** | | | | | |
| --- | --- | --- | --- | --- | --- | --- | --- |
| **Age Group** |  | **Overall** | | **Stay** | | **Switch** | |
|  | **Metric** | **M** | **SD** | **M** | **SD** | **M** | **SD** |
| **12 and under** | *Response Time* | 724.74 | 217.14 | 711.65 | 218.00 | 736.60 | 227.25 |
|  | *Accuracy* | 80% | 9% | 84% | 11% | 76% | 12% |
| **13-17 years** | *Response Time* | 629.64 | 172.89 | 616.10 | 164.81 | 640.68 | 188.55 |
|  | *Accuracy* | 83% | 8% | 88% | 9% | 79% | 11% |
| **18-22 years** | *Response Time* | 500.48 | 139.99 | 494.17 | 139.22 | 502.87 | 142.95 |
|  | *Accuracy* | 88% | 8% | 91% | 8% | 85% | 9% |
| **23-29 years** | *Response Time* | 579.27 | 250.72 | 573.18 | 257.12 | 583.75 | 254.06 |
|  | *Accuracy* | 87% | 7% | 90% | 8% | 83% | 10% |
| **30-39 years** | *Response Time* | 578.56 | 170.28 | 573.75 | 179.24 | 579.46 | 165.40 |
|  | *Accuracy* | 89% | 8% | 92% | 9% | 86% | 10% |
| **40+ years** | *Response Time* | 669.03 | 228.74 | 656.25 | 237.57 | 677.60 | 226.72 |
|  | *Accuracy* | 89% | 7% | 92% | 9% | 86% | 10% |

Table S11. ACE-X mean performance metrics across age groups for Tap and Trace.

|  |  | **Tap and Trace** | | | | | |
| --- | --- | --- | --- | --- | --- | --- | --- |
| **Age Group** |  | **Overall** | | **Tap Only** | | **Tap and Trace** | |
|  | **Metric** | **M** | **SD** | **M** | **SD** | **M** | **SD** |
| **12 and under** | *Response Time* | 729.94 | 212.37 | 616.47 | 127.67 | 864.93 | 363.40 |
|  | *Accuracy* | 95% | 5% | 95% | 7% | 95% | 7% |
| **13-17 years** | *Response Time* | 706.86 | 242.65 | 604.37 | 175.59 | 822.73 | 348.37 |
|  | *Accuracy* | 96% | 4% | 97% | 5% | 96% | 6% |
| **18-22 years** | *Response Time* | 566.22 | 121.18 | 490.41 | 80.66 | 642.80 | 181.66 |
|  | *Accuracy* | 98% | 3% | 96% | 7% | 98% | 3% |
| **23-29 years** | *Response Time* | 672.84 | 286.55 | 587.26 | 269.49 | 762.67 | 326.32 |
|  | *Accuracy* | 98% | 2% | 97% | 4% | 97% | 3% |
| **30-39 years** | *Response Time* | 634.77 | 153.36 | 559.27 | 125.10 | 715.19 | 209.05 |
|  | *Accuracy* | 98% | 3% | 97% | 5% | 97% | 4% |
| **40+ years** | *Response Time* | 774.13 | 377.71 | 636.98 | 199.01 | 919.10 | 575.04 |
|  | *Accuracy* | 97% | 5% | 96% | 7% | 96% | 6% |

Table S12. Estimates of network edge weights of ACE-X task performance.

| **Edge** | | | **Estimate** | **SE** | ***P*** |
| --- | --- | --- | --- | --- | --- |
| Backwards Spatial Span | 🡘 | Forward Spatial Span | 0.33 | 0.04 | < 0.001 |
| Stroop | 🡘 | Forward Spatial Span | 0.11 | 0.04 | 0.007 |
| Boxed | 🡘 | Forward Spatial Span | 0.18 | 0.04 | < 0.001 |
| TaskSwitch | 🡘 | Backwards Spatial Span | 0.17 | 0.04 | < 0.001 |
| Continuous Performance (Impulsive) | 🡘 | Continuous Performance (Sustained) | 0.56 | 0.03 | < 0.001 |
| Tap and Trace | 🡘 | Continuous Performance (Sustained) | 0.15 | 0.04 | < 0.001 |
| TaskSwitch | 🡘 | Continuous Performance (Sustained) | 0.11 | 0.03 | 0.001 |
| Compass | 🡘 | Continuous Performance (Impulsive) | 0.27 | 0.03 | < 0.001 |
| Stroop | 🡘 | Tap and Trace | 0.18 | 0.05 | < 0.001 |
| Boxed | 🡘 | Tap and Trace | 0.17 | 0.04 | < 0.001 |
| Stroop | 🡘 | Compass | 0.13 | 0.04 | 0.001 |
| Flanker | 🡘 | Compass | 0.22 | 0.04 | < 0.001 |
| Boxed | 🡘 | Compass | 0.23 | 0.04 | < 0.001 |
| Boxed | 🡘 | Stroop | 0.31 | 0.04 | < 0.001 |
| TaskSwitch | 🡘 | Stroop | 0.22 | 0.04 | < 0.001 |
| Boxed | 🡘 | Flanker | 0.28 | 0.04 | < 0.001 |
| TaskSwitch | 🡘 | Boxed | 0.22 | 0.04 | < 0.001 |

Table S13. Results of 95% bootstrapped confidence intervals of network edge weights^a.^

| **Edge** | | | **Lower Limit** | **Upper Limit** |
| --- | --- | --- | --- | --- |
| Backwards Spatial Span | 🡘 | Forward Spatial Span | 0.25 | 0.41 |
| Stroop | 🡘 | Forward Spatial Span | 0.04 | 0.19 |
| Boxed | 🡘 | Forward Spatial Span | 0.10 | 0.26 |
| TaskSwitch | 🡘 | Backwards Spatial Span | 0.08 | 0.26 |
| Continuous Performance (Impulsive) | 🡘 | Continuous Performance (Sustained) | 0.49 | 0.62 |
| Tap and Trace | 🡘 | Continuous Performance (Sustained) | 0.08 | 0.23 |
| TaskSwitch | 🡘 | Continuous Performance (Sustained) | 0.05 | 0.18 |
| Compass | 🡘 | Continuous Performance (Impulsive) | 0.18 | 0.34 |
| Stroop | 🡘 | Tap and Trace | 0.07 | 0.29 |
| Boxed | 🡘 | Tap and Trace | 0.09 | 0.26 |
| Stroop | 🡘 | Compass | 0.05 | 0.22 |
| Flanker | 🡘 | Compass | 0.12 | 0.32 |
| Boxed | 🡘 | Compass | 0.15 | 0.31 |
| Boxed | 🡘 | Stroop | 0.22 | 0.39 |
| TaskSwitch | 🡘 | Stroop | 0.14 | 0.30 |
| Boxed | 🡘 | Flanker | 0.19 | 0.35 |
| TaskSwitch | 🡘 | Boxed | 0.13 | 0.30 |
| ^a^Based on a total of 2,500 resampled datasets. | | | | |

Table S14. Means and scaling estimates from network model of ACE-X task performance with edge weights constrained to equality for younger and older adults.

|  | **Means** | | **Scaling** | |
| --- | --- | --- | --- | --- |
|  | **18-39 years** | **40+ years** | **18-39 years** | **40+ years** |
| Forward Spatial Span | 8.22 | 8.00 | 0.79 | 0.71 |
| Backwards Spatial Span | 7.46 | 7.33 | 0.90 | 0.78 |
| Flanker | 2.75 | 2.65 | 0.20 | 0.19 |
| Continuous Performance (Impulsive) | -1.94 | -2.03 | 0.39 | 0.45 |
| Continuous Performance (Sustained) | -2.48 | -2.53 | 0.55 | 0.49 |
| Stroop | 2.55 | 2.37 | 0.22 | 0.23 |
| Boxed | 1.72 | 1.61 | 0.12 | 0.11 |
| Compass | 4.71 | 4.50 | 0.23 | 0.26 |
| TaskSwitch | 2.85 | 2.77 | 0.33 | 0.30 |
| Tap and Trace | -2.43 | -3.28 | 1.12 | 1.41 |

Table S15. Descriptions of flags used as part of exclusionary criteria.

| **Flag** | **Description** |
| --- | --- |
| Duplicated Location | Participant geolocation data exactly matches another participant |
| Timezone Outside the United States^a^ | Participant timezone as pulled from system data is outside of United States |
| Duplicated Internet Protocol Address^a^ | Participant Internet Protocol address exactly matches another participant |
| Age Reported in ACE-X Inconsistent with Birthdate | Participant age self-reported in ACE-X is inconsistent with age as calculated from self-reported date of birth |
| Participant Reported Email with Disposable Email Domain^a^ | Participant self-reported email uses a disposable (temporary) email domain when compared to list of known disposable domains |
| Repeated Sign-ups from Uncommon Email Domain | Consecutive sign-ups of participants using uncommon email domains (email domains other than @gmail.com) |
| Repeated Sign-ups with Same Income | Consecutive sign-ups of participants self-reporting the exact same income |
| Sign-up Occurred within 5 Minutes of Another | Participant signed up for study within five minutes of another participant |
| Basic Response Time Outside of Typical Range for Age Group | Participant basic response time is greater than 4 standard deviations above age-relevant basic response time as compiled from similar studies |
| Repeated Sign-ups from Same Zip Code | Consecutive sign-ups of participants self-reporting the exact same zip code |
| Email Similar to Another Participant | Participant signed up using the same or similar email as another participant |
| Annual Income Greater Than 3 Median Absolute Deviations of Median Income of Sample | Self-reported annual income is greater than 3 median absolute deviations of the median income of the participant sample |
| Annual Income Less Than $10,000 | Self-reported annual income is less than $10,000 |
| Age Reported in ACE-X Inconsistent with Reported Age Group | Age self-reported in ACE-X is inconsistent with self-reported age group in eligibility questionnaire |
| Birthdate Greater than 100 Years in Past | Age as calculated from self-reported birthdate greater than 100 years |
| Repeated Sign-ups Reporting Intersex Gender | Consecutive sign-ups of participants self-reporting themselves as intersex |
| Age Greater than 100 Years Reported in ACE-X | Self-reported age in ACE-X is greater than 100 years |
| Birthdate Inconsistent with Reported Age Group | Participant age group self-reported in eligibility questionnaire is inconsistent with age as calculated from self-reported date of birth |
| Multiple IP Addresses Associated with Participant | Participant questionnaires were completed from more than one Internet Protocol addresses |

^a^Flag used as part of exclusionary criteria.

### Supplementary References

1. Anguera JA, Rowe MA, Volponi JJ, et al. Enhancing attention in children using an integrated cognitive-physical videogame: A pilot study. npj Digit. Med. 2023;6(65). Doi: <https://doi.org/10.1038/s41746-023-00812-z>
2. Anguera JA, Volponi JJ, Simon AJ, Gallen CL, Rolle CE, Anguera-Singla R, Pitsch EA, Thompson CJ, Gazzaley A. Integrated cognitive and physical fitness training enhances attention abilities in older adults. NPJ Aging. Aug 30 2022;8(1):12. doi: 10.1038/s41514-022-00093-y. PMID: 36042247; PMCID: PMC9427998.
3. Anguera JA, Brandes-Aitken AN, Antovich AD, Rolle CE, Desai SS, Marco EJ. A pilot study to determine the feasibility of enhancing cognitive abilities in children with sensory processing dysfunction. PLoS ONE. 2017;12(4):e0172616. doi: <https://doi.org/10.1371/journal.pone.0172616>
4. Anguera JA, Boccanfuso J, Rintoul J, et al. Video game training enhances cognitive control in older adults. Nature. 2013;501:97-101. doi:<https://doi.org/10.1038/nature12486>
5. Bleecker ML, Bolla‐Wilson K, Agnew J, Meyers DA. Simple visual reaction time: Sex and age differences. Developmental Neuropsychology. 1987;3(2):165-172. doi:[10.1080/87565648709540372](https://doi.org/10.1080/87565648709540372)
6. Eckner JT, Kutcher JS, Broglio SP, Richardson JK. Effect of sport-related concussion on clinically measured simple reaction time. Br J Sports Med.Jan 2014;48(2):112-8. doi: 10.1136/bjsports-2012-091579. Epub 2013 Jan 11. PMID: 23314889; PMCID: PMC3732515.
7. Gallen CL, Anguera JA, Gerdes MR, Simon AJ, Cañadas E, Marco EJ. Enhancing neural markers of attention in children with ADHD using a digital therapeutic. PLoS ONE. 2021;16(12): e0261981. doi:<https://doi.org/10.1371/journal.pone.0261981>
8. Lemay S, Bédard MA, Rouleau I, Tremblay PL. Practice effect and test-retest reliability of attentional and executive tests in middle-aged to elderly subjects. The Clinical Neuropsychologist. 2004;18(2):284-302. doi:[10.1080/13854040490501718](https://doi.org/10.1080/13854040490501718)
9. Rolle CE, Anguera JA, Skinner SN, Voytek B, Gazzaley A. Enhancing spatial attention and working memory in younger and older adults. J Cogn Neurosci. 2017;29(9):1483-1497. doi: <https://doi.org/10.1162/jocn_a_01159>
10. Silverman, IW. Simple reaction time: It is not what it used to be. American Journal of Psychology. 2010;123(1):39-50.
11. Stuss DT, Stethem LL, Hugenholtz H, Picton T, Pivik J, Richard MT. Reaction time after head injury: Fatigue, divided and focused attention, and consistency of performance. J Neurol Neurosurg Psychiatry. 1989 Jun;52(6):742-8. doi: 10.1136/jnnp.52.6.742. PMID: 2746267; PMCID: PMC1032026.
12. Zanto TP, Gazzaley A. Fronto-parietal network: Flexible hub of cognitive control. Trends Cogn Sci. 2013 Dec;17(12):602-3. doi: 10.1016/j.tics.2013.10.001. Epub 2013 Oct 12. PMID: 24129332; PMCID: PMC3873155.
13. Zanto TP, Rubens MT, Thangavel A, Gazzaley A. Causal role of the prefrontal cortex in top-down modulation of visual processing and working memory. Nat Neurosci. 2011 May;14(5):656-61. doi: 10.1038/nn.2773. Epub 2011 Mar 27. PMID: 21441920; PMCID: PMC3083493.
14. Ziegler DA, Simon AJ, Gallen CL, et al. Closed-loop digital meditation improves sustained attention in young adults. Nat Hum Behav 2019;3:746–757. doi: https://doi.org/10.1038/s41562-019-0611-9
15. Banich MT, Milham MP, Atchley R, Cohen NJ, Webb A, Wszalek T, Kramer AF, Liang Z-P, Wright A, Shenker J, Magin R. fMRI studies of Stroop tasks reveal unique roles of anterior and posterior brain systems in attentional selection. J Cogn Neurosci. 2000;12:988-1000.
16. Fan J, Flombaum JI, McCandliss BD, Thomas KM, Posner MI (2003): Cognitive and brain consequences of conflict. Neuroimage. 2003;18:42-57.
17. Hasshim N, Downes M, Bate S, Parris BA. Response Time Distribution Analysis of Semantic and Response Interference in a Manual Response Stroop Task. Exp Psychol. 2019 May;66(3):231-238. doi: 10.1027/1618-3169/a000445. PMID: 31266430.
18. Heathcote A, Popiel SJ, Mewhort DJ. Analysis of response time distributions: An example using the Stroop task. Psychological Bulletin. 1991;109(2):340-347. doi:[https://doi.org/10.1037/0033-2909.109.2.340](https://psycnet.apa.org/doi/10.1037/0033-2909.109.2.340)
19. Mead LA, Mayer AR, Bobholz JA, Woodley SJ, Cunningham JM, Hammeke TA, Rao SM. Neural basis of the Stroop interference task: Response competition or selective attention? J Int Neuropsychol Soc. 2002;8:735-742. PMID: 12240737 DOI: 10.1017/s1355617702860015
20. Milham MP, Banich MT, Webb A, Barad V, Cohen NJ, Wszalek T, Kramer AF. The relative involvement of anterior cingulate and prefrontal cortex in attentional control depends on nature of conflict. Brain Res Cogn Brain Res. 2001;12:467-473.
21. Toth AJ, Kowal M, Campbell MJ. The Color-Word Stroop task does not differentiate cognitive inhibition ability among esports gamers of varying expertise. Frontiers in Psychology, 2019;10. https://www.frontiersin.org/articles/10.3389/fpsyg.2019.02852 DOI: 10.3389/fpsyg.2019.02852; ISSN: 1664-1078
22. Eriksen BA, Eriksen CW. Effects of noise letters upon the identification of a target letter in a nonsearch task. Perception & Psychophysics. 1974;16(1):143-149.
23. Haciahmet CC, Frings C, Pastötter B. Target amplification and distractor inhibition: Theta oscillatory dynamics of selective attention in a Flanker task. Cogn Affect Behav Neurosci. 2021 Apr;21(2):355-371. doi: 10.3758/s13415-021-00876-y. Epub 2021 Mar 15. PMID: 33721227; PMCID: PMC8121747.
24. Markiewicz R, Mazaheri A, Krott A. Bilingualism can cause enhanced monitoring and occasional delayed responses in a flanker task. European Journal of Neuroscience. 2023;57(1):129-147. doi:<https://doi.org/10.1111/ejn.15863>
25. Servant M, Logan GD. Dynamics of attentional focusing in the Eriksen flanker task. Attention, Perception, & Psychophysics. 2019;81(8):2710-2721.
26. Stoffels EJ, Van der Molen MW. Effects of visual and auditory noise on visual choice reaction time in a continuous-flow paradigm. Perception & Psychophysics. 1988;44(1):7-14. [https://doi.org/10.3758/BF03207468](https://psycnet.apa.org/doi/10.3758/BF03207468)
27. Wang S, He X, Lu C, et al. Perceptual confusion makes a significant contribution to the conflict effect: Insight from the flanker task and the majority function task. Curr Psychol (2023). <https://doi.org/10.1007/s12144-023-04318-5>
28. Dykstra T, Smith DM, Schumacher EH, Hazeltine E. Measuring task structure with transitional response times: Task representations are more than task sets. Psychon Bull Rev. 2022 Oct;29(5):1812-1820. doi: 10.3758/s13423-021-02035-3. Epub 2022 Apr 8. PMID: 35394643; PMCID: PMC10766293.
29. Erb CD, Touron DR, Marcovitch S. Tracking the dynamics of global and competitive inhibition in early and late adulthood: Evidence from the flanker task. Psychol Aging. 2020 Aug;35(5):729-743. doi: 10.1037/pag0000435. PMID: 32744854.
30. Koch I, Hazeltine E, Petersen G, Weissman DH. Response-repetition costs in task switching do not index a simple response-switch bias: Evidence from manipulating the number of response alternatives. Atten Percept Psychophys. 2023 Nov;85(8):2577-2587. doi: 10.3758/s13414-023-02708-2. Epub 2023 May 5. PMID: 37147509; PMCID: PMC10600293.
31. Kray J, Lindenberger U. Adult age differences in task switching. Psychol Aging. 2000 Mar;15(1):126-47. doi: 10.1037//0882-7974.15.1.126. PMID: 10755295.
32. Mayr U, Kliegl R. Differential effects of cue changes and task changes on task-set selection costs. J Exp Psychol Learn Mem Cogn. 2003 May;29(3):362-72. doi: 10.1037/0278-7393.29.3.362. PMID: 12776747.
33. Monsell S. Task switching. Trends Cogn Sci. 2003 Mar;7(3):134-140. doi: 10.1016/s1364-6613(03)00028-7. PMID: 12639695.
34. Rogers RD, Monsell S. Costs of a predictable switch between simple cognitive tasks. Journal of Experimental Psychology: General. 1995;124(2): 207-231. doi: <https://doi.org/10.1037/0096-3445.124.2.207>
35. Ajana K, Everard G, Lejeune T, Edwards MG. A feature and conjunction visual search immersive virtual reality serious game for measuring spatial and distractor inhibition attention using response time and action kinematics. J Clin Exp Neuropsychol. 2023 May;45(3):292-303. doi: 10.1080/13803395.2023.2218571. Epub 2023 Jun 1. PMID: 37260369.
36. Becker M, McElvany N, Kortenbruck M. Intrinsic and extrinsic reading motivation as predictors of reading literacy: A longitudinal study. Journal of Educational Psychology. 2010;102(4):773-785. doi: https://doi.org/10.1037/a0020084
37. Manley CE, Bauer CM, Bex PJ, Merabet LB. Impaired visuospatial processing in cerebral visual impairment revealed by performance on a conjunction visual search task. British Journal of Visual Impairment. 2023;0(0). doi: <https://doi.org/10.1177/02646196231187550>
38. Merenstein JL, Mullin HA, Madden DJ. Age-related differences in frontoparietal activation for target and distractor singletons during visual search. Atten Percept Psychophys. 2023 Apr;85(3):749-768. doi: 10.3758/s13414-022-02640-x. Epub 2023 Jan 10. PMID: 36627473; PMCID: PMC10066832.
39. Pennington CR, Qureshi AW, Monk RL, Greenwood K, Heim D. Beer? Over here! Examining attentional bias towards alcoholic and appetitive stimuli in a visual search eye-tracking task. Psychopharmacology (Berl). 2019 Dec;236(12):3465-3476. doi: 10.1007/s00213-019-05313-0. Epub 2019 Jul 8. PMID: 31286155; PMCID: PMC6892770.
40. Plude DJ, Doussard-Roosevelt JA. Aging, selective attention, and feature integration. Psychol Aging. 1989 Mar;4(1):98-105. doi: 10.1037/0882-7974.4.1.98. PMID: 2803617.
41. Treisman AM, Gelade G. A feature-integration theory of attention. Cogn Psychol. 1980 Jan;12(1):97-136. doi: 10.1016/0010-0285(80)90005-5. PMID: 7351125.
42. Anguera JA, Jordan JT, Castaneda D, Gazzaley A, Areán PA. Conducting a fully mobile and randomised clinical trial for depression: access, engagement and expense. BMJ Innov. 2016 Jan;2(1):14-21. doi: 10.1136/bmjinnov-2015-000098. PMID: 27019745; PMCID: PMC4789688.
43. Greenberg LM, Waldman ID. Developmental normative data on the test of variables of attention (T.O.V.A.). J Child Psychol Psychiatry. 1993 Sep;34(6):1019-30. doi: 10.1111/j.1469-7610.1993.tb01105.x. PMID: 8408366.
44. Memória CM, Muela HCS, Moraes NC, Costa-Hong VA, Machado MF, Nitrini R, Bortolotto LA, Yassuda MS. Applicability of the Test of Variables of Attention - T.O.V.A in Brazilian adults. Dement Neuropsychol. 2018 Oct-Dec;12(4):394-401. doi: 10.1590/1980-57642018dn12-040009. PMID: 30546850; PMCID: PMC6289477.
45. Arif Y, Spooner RK, Wiesman AI, Embury CM, Proskovec AL, Wilson TW. Modulation of attention networks serving reorientation in healthy aging. Aging (Albany NY). 2020 Jun 24;12(13):12582-12597. doi: 10.18632/aging.103515. Epub 2020 Jun 24. PMID: 32584264; PMCID: PMC7377885.
46. Dukewich KR. Attending to space and time: The effects of attention on multisensory spatial and temporal resolution. Doctoral dissertation, Dalhousie University. 2008.
47. Johnson H, Haggard P. The effect of attentional cueing on conscious awareness of stimulus and response. Exp Brain Res. 2003 Jun;150(4):490-6. doi: 10.1007/s00221-003-1474-9. Epub 2003 May 1. PMID: 12728292.
48. Merritt P, Hirshman E, Wharton W, Devlin J, Stangl B, Bennett S, Hawkins L. Gender differences in selective attention: Evidence from a spatial orienting task. Journal of Vision. 2005;5(8):1000. doi: <https://doi.org/10.1167/5.8.1000>.
49. Perez-Edgar K, Fox NA. A behavioral and electrophysiological study of children's selective attention under neutral and affective conditions. Journal of Cognition and Development. 2005;6(1):89-118. doi: 10.1207/s15327647jcd0601_6
50. Posner MI, Snyder CR, Davidson BJ. Attention and the detection of signals. J Exp Psychol. 1980 Jun;109(2):160-74. PMID: 7381367.
51. Van der Stigchel S, Theeuwes J. The relationship between covert and overt attention in endogenous cuing. Percept Psychophys. 2007 Jul;69(5):719-31. doi: 10.3758/bf03193774. PMID: 17929695.
52. Bherer L, Kramer AF, Peterson MS, Colcombe S, Erickson K, Becic E. Transfer effects in task-set cost and dual-task cost after dual-task training in older and younger adults: further evidence for cognitive plasticity in attentional control in late adulthood. Exp Aging Res. 2008 Jul-Sep;34(3):188-219. doi: 10.1080/03610730802070068. PMID: 18568979; PMCID: PMC2845439.
53. Eversheim U, Bock O. Evidence for processing stages in skill acquisition: a dual-task study. Learn Mem. 2001 Jul-Aug;8(4):183-9. doi: 10.1101/lm.39301. PMID: 11533221; PMCID: PMC311376.
54. Chen ZY, Cowell PE, Varley R, Wang YC. A cross-language study of verbal and visuospatial working memory span. J Clin Exp Neuropsychol. 2009 May;31(4):385-91. doi: 10.1080/13803390802195195. PMID: 18720176.
55. Corsi PM. Human memory and the medial temporal region of the brain. Dissertation Abstracts International. 1973;34(2-B), 891.
56. Farrell Pagulayan K, Busch RM, Medina KL, Bartok JA, Krikorian R. Developmental normative data for the Corsi Block-tapping task. J Clin Exp Neuropsychol. 2006 Aug;28(6):1043-52. doi: 10.1080/13803390500350977. PMID: 16822742.
57. Hazarika J, Dasgupta R. Neural correlates of action video game experience in a visuospatial working memory task. Neural Comput & Applic. 2020;32:3431–3440. doi: https://doi.org/10.1007/s00521-018-3713-9
58. Kessels RP, van den Berg E, Ruis C, Brands AM. The backward span of the Corsi Block-Tapping Task and its association with the WAIS-III Digit Span. Assessment. 2008 Dec;15(4):426-34. doi: 10.1177/1073191108315611. Epub 2008 May 15. PMID: 18483192.
59. Mammarella IC, Cornoldi C. Difficulties in the control of irrelevant visuospatial information in children with visuospatial learning disabilities. Acta Psychol (Amst). 2005 Mar;118(3):211-28. doi: 10.1016/j.actpsy.2004.08.004. PMID: 15698821.
60. Siddi S, Preti A, Lara E, et al. Comparison of the touch-screen and traditional versions of the Corsi block-tapping test in patients with psychosis and healthy controls. BMC Psychiatry 2020;20:329. doi: https://doi.org/10.1186/s12888-020-02716-8
61. Vandierendonck A, Kemps E, Fastame MC, Szmalec A. Working memory components of the Corsi blocks task. Br J Psychol. 2004 Feb;95(Pt 1):57-79. doi: 10.1348/000712604322779460. PMID: 15005868.
62. de Paula JJ, Malloy-Diniz LF, Romano-Silva MA. Reliability of working memory assessment in neurocognitive disorders: A study of the Digit Span and Corsi Block-Tapping tasks. Braz J Psychiatry. 2016 Jul-Sep;38(3):262-3. doi: 10.1590/1516-4446-2015-1879. PMID: 27579598; PMCID: PMC7194262.
63. Furley P, Memmert D. Differences in spatial working memory as a function of team sports expertise: the Corsi Block-tapping task in sport psychological assessment. Percept Mot Skills. 2010 Jun;110(3 Pt 1):801-8. doi: 10.2466/PMS.110.3.801-808. PMID: 20681333.
